# Comparison of robot-assisted versus fluoroscopy-guided transforaminal lumbar interbody fusion (TLIF) for lumbar degenerative diseases: a systematic review and meta-analysis of randomized controlled trails and cohort studies

**DOI:** 10.1186/s13643-024-02600-6

**Published:** 2024-07-05

**Authors:** Jianbin Guan, Ningning Feng, Xing Yu, Kaitan Yang

**Affiliations:** 1 Honghui-Hospital, Xi’an Jiaotong University, Xi’an, 710054 China; 2 Shannxi Key Laboratory of Spine Bionic Treatment, Xi’an, China; 3 Dongzhimen Hospital Affiliated to Beijing University of Chinese Medicine, Beijing, 100700 China; 4 Truma Rehabilitation Department, Honghui-Hospital, Xi’an Jiaotong University, Xi’an, 710054 China

**Keywords:** Robot-assisted, Transforaminal lumbar interbody fusion, Fluoroscopy-guided, Meta-analysis, Pedicle screw placement

## Abstract

**Background:**

As an emerging technology in robot-assisted (RA) surgery, the potential benefits of its application in transforaminal lumbar interbody fusion (TLIF) lack substantial support from current evidence.

**Objective:**

We aimed to investigate whether the RA TLIF is superior to FG TLIF in the treatment of lumbar degenerative disease.

**Methods:**

We systematically reviewed studies comparing RA versus FG TLIF for lumbar degenerative diseases through July 2022 by searching PubMed, Embase, Web of Science, CINAHL (EBSCO), Chinese National Knowledge Infrastructure (CNKI), WanFang, VIP, and the Cochrane Library, as well as the references of published review articles. Both cohort studies (CSs) and randomized controlled trials (RCTs) were included. Evaluation criteria included the accuracy of percutaneous pedicle screw placement, proximal facet joint violation (FJV), radiation exposure, duration of surgery, estimated blood loss (EBL), and surgical revision. Methodological quality was assessed using the Cochrane risk of bias and ROBINS-I Tool. Random-effects models were used, and the standardized mean difference (SMD) was employed as the effect measure. We conducted subgroup analyses based on surgical type, the specific robot system used, and the study design. Two investigators independently screened abstracts and full-text articles, and the certainty of evidence was graded using the GRADE (Grading of Recommendations Assessment, Development and Evaluation) approach.

**Results:**

Our search identified 539 articles, of which 21 met the inclusion criteria for quantitative analysis. Meta-analysis revealed that RA had 1.03-folds higher “clinically acceptable” accuracy than FG (RR: 1.0382, 95% CI: 1.0273–1.0493). And RA had 1.12-folds higher “perfect” accuracy than FG group (RR: 1.1167, 95% CI: 1.0726–1.1626). In the case of proximal FJV, our results indicate a 74% reduction in occurrences for patients undergoing RA pedicle screw placement compared to those in the FG group (RR: 0.2606, 95%CI: 0.2063- 0.3293). Seventeen CSs and two RCTs reported the duration of time. The results of CSs suggest that there is no significant difference between RA and FG group (SMD: 0.1111, 95%CI: -0.391–0.6131), but the results of RCTs suggest that the patients who underwent RA-TLIF need more surgery time than FG (SMD: 3.7213, 95%CI: 3.0756–4.3669). Sixteen CSs and two RCTs reported the EBL. The results suggest that the patients who underwent RA pedicle screw placement had fewer EBL than FG group (CSs: SMD: -1.9151, 95%CI: -3.1265–0.7036, RCTs: SMD: -5.9010, 95%CI: -8.7238–3.0782). For radiation exposure, the results of CSs suggest that there is no significant difference in radiation time between RA and FG group (SMD: -0.5256, 95%CI: -1.4357–0.3845), but the patients who underwent RA pedicle screw placement had fewer radiation dose than FG group (SMD: -2.2682, 95%CI: -3.1953–1.3411). And four CSs and one RCT reported the number of revision case. The results of CSs suggest that there is no significant difference in the number of revision case between RA and FG group (RR: 0.4087,95% CI 0.1592–1.0495). Our findings are limited by the residual heterogeneity of the included studies, which may limit the interpretation of the results.

**Conclusion:**

In TLIF, RA technology exhibits enhanced precision in pedicle screw placement when compared to FG methods. This accuracy contributes to advantages such as the protection of adjacent facet joints and reductions in intraoperative radiation dosage and blood loss. However, the longer preoperative preparation time associated with RA procedures results in comparable surgical duration and radiation time to FG techniques. Presently, FG screw placement remains the predominant approach, with clinical surgeons possessing greater proficiency in its application. Consequently, the integration of RA into TLIF surgery may not be considered the optimal choice.

**Systematic review registration:**

PROSPERO CRD42023441600.

**Supplementary Information:**

The online version contains supplementary material available at 10.1186/s13643-024-02600-6.

## Introduction

Since the first report of transforaminal lumbar interbody fusion (TLIF) for the treatment of lumbar spondylolisthesis by Harms and Rolinger et al. [1] in 1982, TLIF has progressively evolved into a standard surgical procedure for addressing lumbar degenerative diseases [2]. Subsequently, Foley et al. [3] further advanced TLIF by introducing the minimally invasive technique (Wiltse technique). This breakthrough facilitated the initial adoption of minimally invasive surgery (MIS) in TLIF, leading to decreased surgical trauma, accelerated recovery, and an overall alleviation of the patient's daily life burden. Nevertheless, TLIF is not without its drawbacks, including prolonged surgical time and a steep learning curve. The duration of the surgery frequently hinges on the surgeon's proficiency in mastering technical skills [4, 5]. The restricted operating field frequently results in imprecise screw placement, often requiring additional corrective surgeries. To guarantee optimal accuracy in screw placement, real-time fluoroscopic examination is typically considered essential throughout the procedure. Consequently, the potential for excessive radiation exposure during MIS-TLIF remains a significant concern [6, 7]. Undoubtedly, whether it is fluoroscopy-guided (FG) TLIF or MIS-TLIF, the most critical aspect of the surgical procedure is the swift and accurate placement of pedicle screws. This objective is paramount in reducing surgical time, minimizing intraoperative bleeding, enhancing surgical outcomes, lowering the rate of revision surgeries, and mitigating radiation exposure. Therefore, achieving expedient and precise placement of pedicle screws remains an urgent concern in TLIF.

The integration of robotic technology into spine surgery has offered a solution for achieving accurate and efficient pedicle screw placement. Robotics can assist surgeons in precise navigation and access to critical anatomical structures during spinal surgery, leveraging 3D imaging. Furthermore, the employment of surgical robots for pedicle screw placement ensures both safety and accuracy, while also minimizing the surgeon's exposure to intraoperative radiation. However, at present, FG techniques persist as the predominant method for screw insertion in TLIF, with surgeons exhibiting greater proficiency in its application. As a nascent robot-assisted (RA) technology, the potential superiority of its application in TLIF surgery has not yet been substantiated by relevant evidence. Furthermore, opting for RA procedures in TLIF imposes a heightened financial burden on patients compared to traditional FG-TLIF. Consequently, the suitability of integrating RA technology into TLIF surgery remains uncertain [8, 9]. In order to examine the potential advantages of RA in terms of screw placement accuracy and its ability to address the limitations of FG in TLIF, we conducted a systematic review and meta-analysis.

## Methods

This systematic review and meta-analysis are performed based on the guidance of the Preferred Reporting Items for Systematic Reviews and Meta-Analysis (PRISMA, Text 1) and Cochrane Handbook for Systematic Reviews of Interventions [10, 11]. No ethical approval and patient consent are required because all analyses are based on previous published studies. The full protocol for this study is available in the supplementary material (Text 2). Literature search, data extraction, data synthesis, and quality assessment were conducted by at least two professional reviewers. The review protocols were retrospective registered on PROSPERO (International Prospective Register of Systematic Reviews, No. CRD42023441600). Our study was conducted retrospectively on July 12, 2022. The retrospective registration in no way compromises the quality, validity, or integrity of the research findings presented in this manuscript. All research procedures, data collection, and data analysis were carried out systematically and well-documented, ensuring the reliability and reproducibility of our results.

### Search strategy and selection criteria

We systematically searched several databases, including PubMed, Excerpta Medical database (Embase), Web of Science, CINAHL (EBSCO), China National Knowledge Infrastructure (CNKI), WanFang Database (WanFang), China Science and Technology Journal Database (VIP), and the Cochrane Library, from inception to July 2022 using the following keywords combined with MeSH terms: 'robot-assisted,' 'fluoroscopy-assisted,' 'lumbar surgery,' 'spinal surgery,' 'transforaminal lumbar interbody fusion,' and 'minimally invasive surgery,' 'TLIF,' 'MIS-TLIF,' 'RA,' 'FG,' and 'lumbar degenerative diseases.' Search terms were combined using the Boolean operators 'AND' or 'OR.' Furthermore, the reference lists of manuscripts were also hand-searched to ensure that some studies, which were not identified by our original search, were also included in the present study. The complete search strategies were shown in Supplementary material 1.

We incorporated all types of relevant studies, encompassing randomized controlled trials (RCTs) as well as prospective and retrospective cohort studies (CSs). The study population comprised patients diagnosed with degenerative lumbar spinal diseases, such as spondylolisthesis and lumbar spinal stenosis, who underwent treatment via TLIF. In the included studies, the intervention group must be RA TLIF, and the control group is FG TLIF (Table 1). The following exclusion criteria were used: (1) studies with insufficient data; (2) cadaveric and animal studies; (3) sample size per arm < 10 participants; and (4) patients with other treatment. Moreover, there were no language restrictions.

**Table 1:** PICO question breakdown for interventions in degenerative lumbar spinal diseases treatment

Participant	Intervention	Comparator	Outcome
• Patient with degenerative lumbar spinal diseases, such as spondylolisthesis, lumbar spinal stenosis • Patient who underwent TLIF	Robot-assisted TLIF	Fluoroscopy-guided TLIF	• Accuracy of percutaneous pedicle screw placement and proximal FJV • Perioperative parameter such as radiation exposure, duration of surgery and EBL • Other outcome such as revision case

*Abbreviations*: *TLIF* Transforaminal interbody lumbar fusion, *FJV* Facet joint violation, *EBL* Estimated blood loss, *PICO* Population/Participant, Intervention, Comparison, Outcome

### Data extraction and synthesis

The two reviewers (JB.G and NN.F) extracted data independently using a standardized form. The following factors were recorded when the information in the reviewed articles was available: first author, year, participants and surgery, type of surgery, type of robot system, sample size, age, sex, study design, intra-pedicular accuracy, proximal facet joint violation (FJV), duration of surgery, estimated blood loss (EBL), radiation time and dose, and revision case. Any disagreements between the reviewers (JB.G and NN.F) were resolved through discussion. In case of insolvable discrepancies, a third reviewer (KT.Y) acted as an arbitrator.

The primary outcomes include the accuracy of percutaneous pedicle screw placement and the occurrence of proximal facet joint violation (FJV). For intra-pedicular accuracy, the positions of pedicle screws were classified using the Gertzbein and Robbins criteria [12]. Grade A represents an intra-pedicular screw without breaching the cortical layer of the pedicle. Grade B refers to a screw that breaches the cortical layer of the pedicle but does not exceed it laterally by more than 2 mm. Grades C and D indicate penetration of less than 4 mm and 6 mm, respectively (indicated by arrows). Grade E is assigned to screws (indicated by arrows) that either do not pass through the pedicle or, at any point in their intended intra-pedicular course, breach the cortical layer of the pedicle in any direction by more than 6 mm. Proximal FJV was assessed according to the violation grade proposed by Babu et al. [13]. The grading system for violations was as follows: Grade 0 represented pedicle screws that did not encroach on the facet joint. Grade 1 defined pedicle screws that violated the facet joint surface by ≤ 1 mm. Grade 2 represented pedicle screws that clearly violated the facet joint. The secondary outcomes include radiation time and dose (duration of radiation exposure and amount of radiation administered during the surgery), duration of surgery (total time required for the surgical procedure), estimated blood loss (EBL, an estimation of the amount of blood lost during surgery), and surgical revision (instances where revision surgery was required due to complications or issues with the initial pedicle screw placement).

The minimally important difference (MID) is the smallest amount of improvement in a treatment outcome that patients would recognize as important. For Proximal FJV, a lower grade is better, and the MID is Grade A. Regarding intra-pedicular accuracy, the MID of Grade 0 represents perfect intra-pedicular localization with no cortical breach. Any deviation from perfect intra-pedicular localization (i.e., any grade higher than 0) would be considered clinically meaningful. As for all secondary outcomes, there are no articles discussing the MID for them, but lower values are considered better.

Two investigators independently selected articles based on the criteria described above. The full text was scanned to determine whether the articles met the inclusion criteria. Disagreements were resolved through discussion until a consensus was reached. If no consensus was reached, a third investigator was consulted.

In this study, our primary objectives included assessing the accuracy of percutaneous pedicle screw placement, proximal FJV, radiation exposure, duration of surgery, EBL, and the necessity for surgical revision. We selected these outcomes based on their clinical relevance to spinal surgery and their alignment with the specific research questions we aimed to address. However, we must acknowledge that the manuscript does not include several outcomes that were initially planned in the study protocol, such as the length of hospital stay, VAS for leg pain and back pain, and the Oswestry Disability Index. The decision to exclude these outcomes was made after careful consideration of data availability and their alignment with the primary research objectives. The omission of these outcomes does not compromise the validity of our findings concerning the primary objectives mentioned above. We believe that focusing on these specific outcomes provided a more focused and in-depth analysis of the key aspects of our study.

### Risk of bias and quality of evidence

The methodological quality of the included studies was evaluated using the Cochrane Risk of Bias Tool for randomized controlled trials (RCTs) and the Risk of Bias in Non-Randomized Studies—of Interventions (ROBINS-I) Tool for non-RCTs. Two researchers conducted the assessments independently. In instances of disagreement, a third researcher made the final decision. The ROBINS-I tool encompasses an evaluation of bias risks related to confounding factors (such as insufficient information on the number of operation levels, baseline health status, surgeon experience, patient selection criteria, or center-specific factors), participant selection, intervention classification, deviations from the intended intervention, missing data, outcome measurement, and the selection of reported results [14].

The Grading of Recommendations Assessment, Development, and Evaluation (GRADE) tool was used to assess the overall quality and strength of available evidence. With the use of this approach, evidence is classified as “very low,” “low,” “moderate,” or “high” quality. Evidence from RCTs receives a default grade of “high” quality but may be downgraded based on prespecified criteria. Reasons for downgrading include risk of bias (assessed through the Cochrane Risk of Bias tool and ROBIN-I tool), inconsistency (substantial unexplained interstudy heterogeneity; I^2^ ≥ 50%, *P* < 0.10), indirectness (presence of factors that limited the generalizability of the results), imprecision (the 95% CI for effect estimates were wide or crossed a minimally important difference for benefit or harm), and publication bias (significant evidence of small-study effects).

### Subgroup analysis

We conducted subgroup analyses if there were 2 or more studies in a given subgroup and performed tests of interaction to establish whether the subgroups differed significantly from one another. We assessed the credibility of significant subgroup effects (*P* < 0.05) using previously suggested criteria. Subgroup analyses was performed for type of surgery, type of robot system and study design.

### Statistical analysis

We assess standard mean difference (SMD) with 95% confidence interval (CI) for continuous outcomes and risk ratio (RR) with 95% CI for dichotomous outcomes. Random models were used for all analyses and not to rely on (arbitrary) cut of values for heterogeneity. The rationale for this is that studies on these patient populations cannot be assumed to have one true mean estimate. Statistical heterogeneity was assessed with the Q-test and the I^2^ statistic. I^2^ values of 25%, 50%, and 75% were considered to indicate low, moderate, and high heterogeneity, respectively [15]. If more than 10 studies were available for a particular comparison, we used funnel plots to determine publication bias. Sensitivity analysis using the trim and fill method is employed to assess the stability of the meta-analysis results [16]. If there is little difference in the funnel plot before and after the trim and fill method, it indicates that the results are stable and highly reliable. And missing values were handled, and imputation methods (mean SD from similar studies) was used.

Data were analyzed with the open-source, meta-analysis software OpenMeta-Analyst, which uses R as the underlying statistical engine [17]. All figures were generated using RStudio.

## Results

### Search results and trial characteristics

**Fig. 1:**
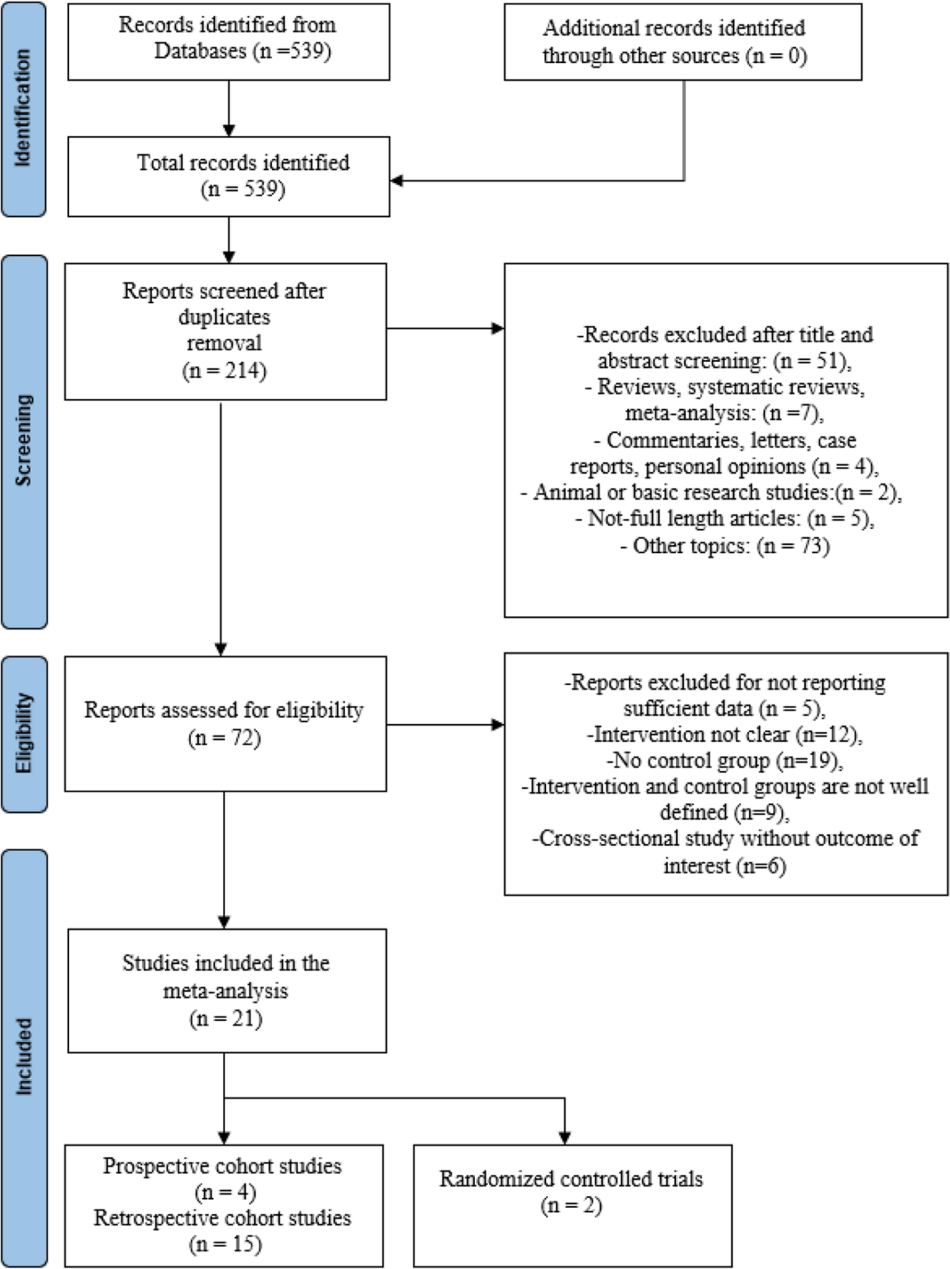
Preferred Reporting Items for Systematic reviews and Meta-Analyses (PRISMA) flow chart of the selection process. (The PRISMA 2020 statement: an updated guideline for reporting systematic reviews) [38]

Title and abstract literature review yielded 539 articles, of which 72 met the inclusion criteria for full text review (Fig. 1). References of 7 systematic reviews found through our online search were also reviewed for relevant articles. A final 21 articles met the inclusion criteria for quantitative analysis. Among them, there were 2 randomized controlled trials (RCTs) [18, 19] and 19 CSs [8, 20–37]. Among the twenty-one studies, six of included studies used Renaissance™ system [20, 21, 26, 27, 30, 32], eleven of included studies used TiRobot system [18, 19, 22–25, 28, 34–37], three of included studies used ROSA™ system [8, 31, 33] and one of included studies used Mazor X Robot system [29]. In terms of surgical type, five of included studies applied the robot system in TLIF surgery [20, 22–24, 30] and sixteen of included studies used the robot system in MIS-TLIF surgery [8, 18, 19, 21, 25–29, 31–37]. Characteristics of included studies are summarized in Table 2.

**Table 2:** The characteristic of the included studies

Authors	Year	Participants and Surgery	Robot System (Company)	Sample Size(I/C) (screw)	Age(years) (mean ± SD) or (range)	Sex(female) n (%)	Outcome	Study design
Schatol B [20]	2014	Lumbar degenerative disease with TLIF and decompressive	Renaissance™ (Mazor Robotics Ltd, Caesarea, Israel)	55(244)/40(163)	52(27–83)/58(23–82)	29(52.7)/28(70)	①③④	Retrospective CS
Yang JS [21]	2019	Lumbar spondylolisthesis who underwent MIS-TLIF	Renaissance™ (Mazor Robotics Ltd, Caesarea, Israel)	30(130)/30(130)	54.1 ± 7.7/55.1 ± 8.1	14(46.7)/12(40)	①②	Retrospective CS
Zhang Q [22] A	2019	Lumbar degenerative disease who underwent TLIF	TiRobot (TINAVI Medical Technologies Co. Ltd, Beijing, China)	43(176)/44(204)	56.7 ± 12.5/60.2 ± 10.9	31(72.1)/26(59.1)	①②③④⑤⑥⑦	Retrospective CS
Zhang Q [23] B	2019	Lumbar degenerative disease who underwent TLIF	TiRobot (TINAVI Medical Technologies Co. Ltd, Beijing, China)	50(100)/50(100)	54.6 ± 11.1/55.6 ± 12.8	33(66)/29(58)	①②③④⑤⑥	Prospective CS
Mao JP [24]	2019	Spondylolisthesis and lumbar spinal stenosis who underwent TLIF	TiRobot (TINAVI Medical Technologies Co. Ltd, Beijing, China)	57(234)/59(278)	55.1 ± 12.4/59.2 ± 11.7	39(68.4)/34(57.6)	①③④⑤⑥	Prospective CS
Jiang SD [25]	2020	Single degenerative lumbar disorders scheduled to MIS-TLIF	TiRobot (TINAVI Medical Technologies Co. Ltd, Beijing, China)	12(48)/32(80)	N/A	N/A	①③④	Retrospective CS
Zhao XF [26]	2020	Spondylolisthesis and lumbar spinal stenosis who underwent MIS-TLIF	Renaissance™ (Mazor Robotics Ltd, Caesarea, Israel)	45(216)/48(238)	54.2 ± 11.5/55.4 ± 10.3	24(53.3)/25(52.1)	①③④	Prospective CS
Wang TY [27]	2021	Consecutive adult patients undergoing MIS-TLIF	Renaissance™ (Mazor Robotics Ltd, Caesarea, Israel)	13/32	68.5/66.7	9(69.2)/20(63.6)	③	Retrospective CS
Chen XY [28]	2021	Consecutive patients who underwent one-level RA MIS-TLIF and one-level open TLIF	TiRobot (TINAVI Medical Technologies Co. Ltd, Beijing, China)	52(208)/52(208)	57.9 ± 12.6/58.1 ± 9.9	20(38.4)/21(40.3)	①③④⑤	Retrospective CS
Cui GY [18]	2021	Spondylolisthesis who underwent MIS-TLIF	TiRobot (TINAVI Medical Technologies Co. Ltd, Beijing, China)	23(92)/25(100)	51.3 ± 9.8/54.1 ± 10.2	19(82.6)/19(76)	①③④⑦	RCT
De Biase G [29]	2021	Lumbar degenerative disease who underwent MIS-TLIF	Mazor X Robot (Medtronic, Dublin, Ireland)	52(228)/49(202)	56 ± 11.7/58.7 ± 10.5	24(42.8)/26(53)	③④⑥⑦	Retrospective CS
Passias PG [8]	2021	Lumbar degenerative disease who underwent MIS-TLIF	ROSA™ Robot System Assistant software	120/120	58.1 ± 13.3/57.6 ± 12.1	N/A	③④⑦	Retrospective CS
Chang M [19]	2022	L4/5 spinal stenosis and Meyerding grade I spondylolisthesis who underwent MSI-TLIF	TiRobot (TINAVI Medical Technologies Co. Ltd, Beijing, China)	26/32	N/A	N/A	③④	RCT
Lai YP [30]	2022	Lumbar degenerative disease who underwent TLIF	Renaissance™ (Mazor Robotics Ltd, Caesarea, Israel)	29/79	66(60–76.5)/64(54.5–71)	15(51.7)/46(58.2)	③④⑦	Retrospective CS
Shafi KA [31]	2022	Spinal stenosis, with or without spondylolisthesis, or recurrent disc herniation, who underwent MIS-TLIF	ROSA™ Robot System Assistant software	92(305)/130(1376)	58.5 ± 12.9/59.5 ± 12.1	45(48.9)/61(46.9)	①	Retrospective CS
Hou HT [32]	2022	Consecutive patients who underwent one-level MIS-TLIF	Renaissance™ (Mazor Robotics Ltd, Caesarea, Israel)	49(196)/49(196)	63.8 ± 12.3/62.9 ± 13.2	25(51)/26(53)	①③④⑤⑥⑦	Retrospective CS
Lin MC [33]	2022	Lumbar degenerative disease who underwent MIS-TLIF	ROSA™ Robot System Assistant software	75(364)/149(682)	65.4 ± 10/62.7 ± 12.6	45(60)/85(57)	③④	Retrospective CS
Li T [34]	2022	Spondylolisthesis and lumbar spinal stenosis who underwent MIS-TLIF	TiRobot (TINAVI Medical Technologies Co. Ltd, Beijing, China)	33(132)/39(156)	53.9 ± 5.1/54.6 ± 5.2	13(39.4) /16(41)	①③④⑤⑥	Retrospective CS
Wang Z [35]	2023	Spondylolisthesis and lumbar spinal stenosis who underwent MIS-TLIF	TiRobot (TINAVI Medical Technologies Co. Ltd, Beijing, China)	73(334)/54(246)	56.6 ± 8.7/56.8 ± 10.1	38(52.1)/27(50)	①②③④	Prospective CS
Wang LL [36]	2023	Spondylolisthesis and lumbar spinal stenosis who underwent MIS-TLIF	TiRobot (TINAVI Medical Technologies Co. Ltd, Beijing, China)	61(274)/62(282)	57.5 ± 8.7/57.7 ± 9.2	45(73.8)/41(66.1)	①②③④	Retrospective CS
Li T [37]	2023	Single degenerative lumbar disorders scheduled to MIS-TLIF	TiRobot (TINAVI Medical Technologies Co. Ltd, Beijing, China)	27(108)/26(104)	53.7 ± 5.9/54 ± 6.4	15(55.6)/16(61.5)	①③④⑤⑥	Retrospective CS

Outcome: ①Gertzbein-Rrobbins scale ②Proximal facet joint violation ③Duration of surgery ④Estimated blood loss ⑤Radiation time ⑥Radiation dose ⑦Revision case

*Abbreviations*: *RA* Robot-assisted, *FG* Fluoroscopy-guided, *MIS* Minimal invasive surgery, *TLIF* Transforaminal interbody lumbar fusion, *RCT* Randomized controlled trial, *CS* Cohort study

### Primary outcome

#### The accuracy of pedicle screw placement

The comparison of the accuracy of pedicle screw placement between RA-and FG-TLIF according to Gertzbein and Robbins criteria in fourteen CSs. If any portion of the screw was ≤ 3 mm outside the pedicle (Grade A + B), we categorized them as “clinically acceptable” accuracy. And the portion of the screw was not deviation (Grade A), we categorized them as “perfect” accuracy.

#### The “clinically acceptable” accuracy

**Fig. 2:**
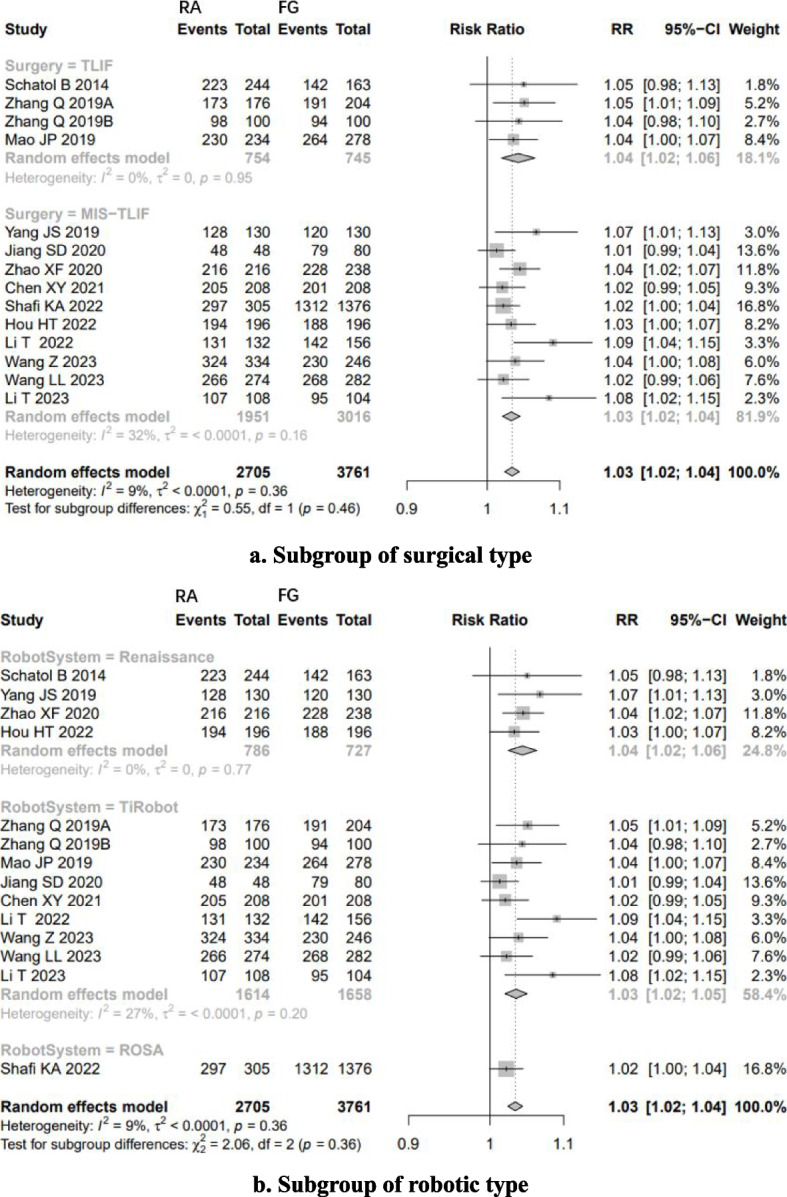
Pooled Analysis of Pedicle Screw Insertion “clinically acceptable” Accuracy. **a** Subgroup of surgical type. **b** Subgroup of robotic type

**Fig. 3:**
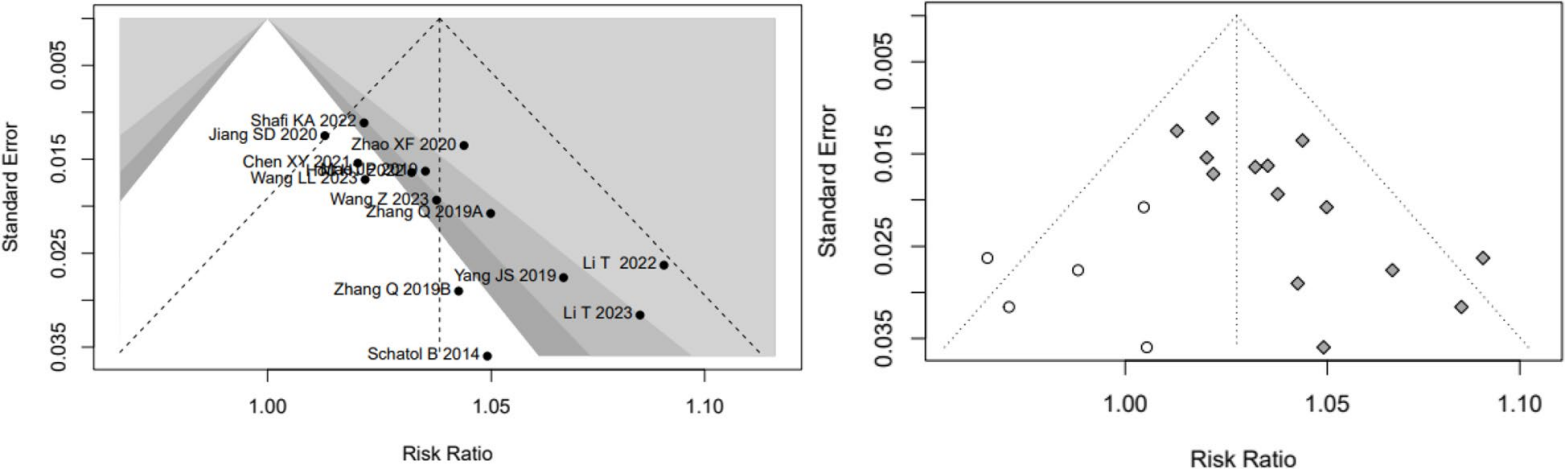
Funnel plot of CSs comparing the “clinically acceptable” accuracy of pedicle screw placement between RA and FG TLIF (left). And the shape of funnel plot after trim-and-fill method (right). No funnel plot of RCTs has been included as there were fewer than 10 RCTs

Low-quality evidence from fourteen CSs [20–26, 28, 31, 32, 34–37] (Table 3), reported a significant difference in “clinically acceptable” accuracy between RA- and FG-TLIF, and RA had 1.03-folds higher “clinically acceptable” accuracy than FG (RR: 1.0382, 95% CI: 1.0273–1.0493, z = 6.96, I^2^ = 9%, *p* < 0.0001, Fig. 2). The funnel plot demonstrates a mostly symmetrical distribution, and minimal changes are observed after applying the trim-and-fill method. This indicates a high level of confidence in the result (Fig. 3).

**Table 3:** GRADE Assessment of Included Studies

Outcomes	Conclusion statement	Relative effect (95% CI)	No. of participants (no. of studies)	Certainty in the evidence (Grade)	Comments
The “clinically acceptable” accuracy of pedicle screw placement	RA had 1.03-folds higher “clinically acceptable” accuracy than FG	RR 1.03 (1.0273–1.0493)	6466 pedicle screws (14 CSs)	⨁⨁◯◯ Low	Rated up for positive association and rated down for risk of bias
The “perfect” accuracy of pedicle screw placement	RA had 1.12-folds higher “perfect” accuracy than FG group	RR 1.12 (1.0726–1.1626)	6466 pedicle screws (14 CSs) 192 pedicle screws (1 RCT)	⨁⨁◯◯ Low	Rated up for positive association and rated down for great heterogeneity of (I^2^ = 75%) and risk of bias The direction of the effect is supported by 1 RCT
Proximal facet joint violation	The patients who underwent RA pedicle screw placement had 74% fewer proximal-facet joint violation than the FG group	RR 0.26 (0.2063- 0.3293)	1881 pedicle screws (5 CSs)	⨁⨁◯◯ Low	Rated up for positive association and rated down for risk of bias
Duration of surgery	CSs suggest that there is no significant difference between RA and FG group, however, the results of RCTs suggest that the patients who underwent RA pedicle screw placement need more surgery time than FG group	SMD_RCTs_ 3.7213 (3.0756–4.3669)	1776 patients (7 cohort studies) 106 (2 RCTs)	⨁◯◯◯ Very low	Rated down for great heterogeneity of CSs (I^2^ = 93%) and rated down for risk of bias No analysis of publication bias The indirection of the effect is supported by 2 RCTs
Estimated blood loss	The patients who underwent RA pedicle screw placement had fewer estimated blood loss than FG group	SMD_CSs_ -1.9151 (-3.1265–0.7036) SMD_RCTs_ -5.9010 (-8.7238–3.0782)	1507 patients (17 cohort studies) 106 patients (2 RCTs)	⨁⨁◯◯ Low	Rated down for great heterogeneity (I^2^ = 98% and 88%) and risk of bias, rated up for positive The direction of the effect is supported by 2 RCTs
Radiation time	There is no significant difference in radiation time between RA and FG group	SMD -0.5256 (-1.4357–0.3845)	730 patients (7 cohort studies)	⨁◯◯◯ Very low	Great heterogeneity (I^2^ = 98%) and moderate risk of bias No analysis of publication bias
Radiation dose	The patients who underwent RA pedicle screw placement had fewer radiation dose than FG group	SMD -2.2682 (-3.1953–1.3411)	730 patients (7 cohort studies)	⨁◯◯◯ Very low	Great heterogeneity (I^2^ = 94%) No analysis of publication bias
Surgical revision	There is no significant difference in the number of surgical revisions between RA and FG group	RR 0.4087 (0.1592–1.0495)	583 patients (4 cohort studies) 192patients (1 RCTs)	⨁⨁◯◯ Low	Rated up for positive association and rated down for risk of bias The direction of the effect is supported by 1 RCT

GRADE Working Group grades of evidence

High quality: Further research is very unlikely to change our confidence in the estimate of effect

Moderate quality: Further research is likely to have an important impact on our confidence in the estimate of effect and may change the estimate

Low quality: Further research is very likely to have an important impact on our confidence in the estimate of effect and is likely to change the estimate

Very low quality: We are very uncertain about the estimate

Subgroup analysis based on surgical type showed that RA had higher “clinically acceptable” accuracy than FG both in TLIF (RR: 1.04, 95% CI: 1.02–1.06, I^2^ = 0%, *p* < 0.05, Fig. 2a) and MIS-TLIF (RR: 1.03, 95% CI: 1.02–1.04, I^2^ = 32%, *p* < 0.05, Fig. 2a).

Subgroup analysis based on robotic type showed that Renaissance™ system, TiRobot and ROSA ™ system assisted TLIF have higher “clinically acceptable” accuracy than FG-TLIF (Fig. 2b).

#### The “perfect” accuracy

**Fig. 4:**
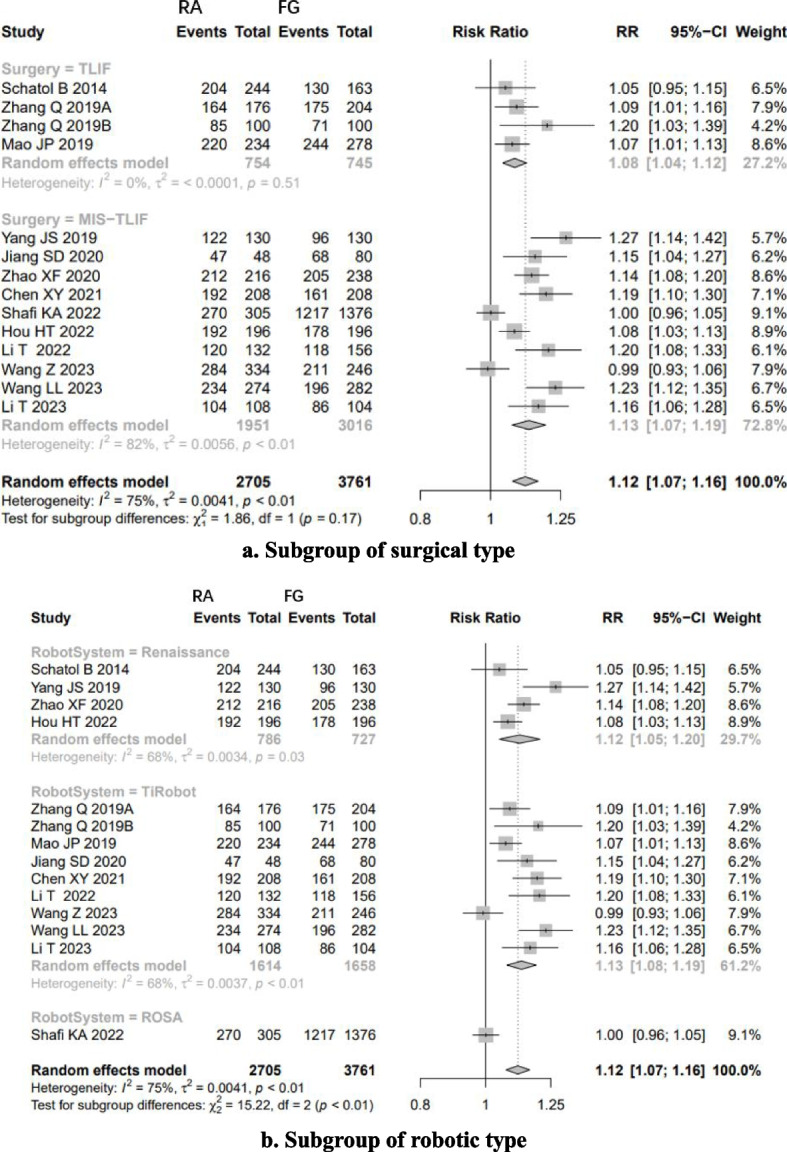
Pooled Analysis of Pedicle Screw Insertion “perfect” Accuracy. **a** Subgroup of surgical type. **b** Subgroup of robotic type

**Fig. 5:**
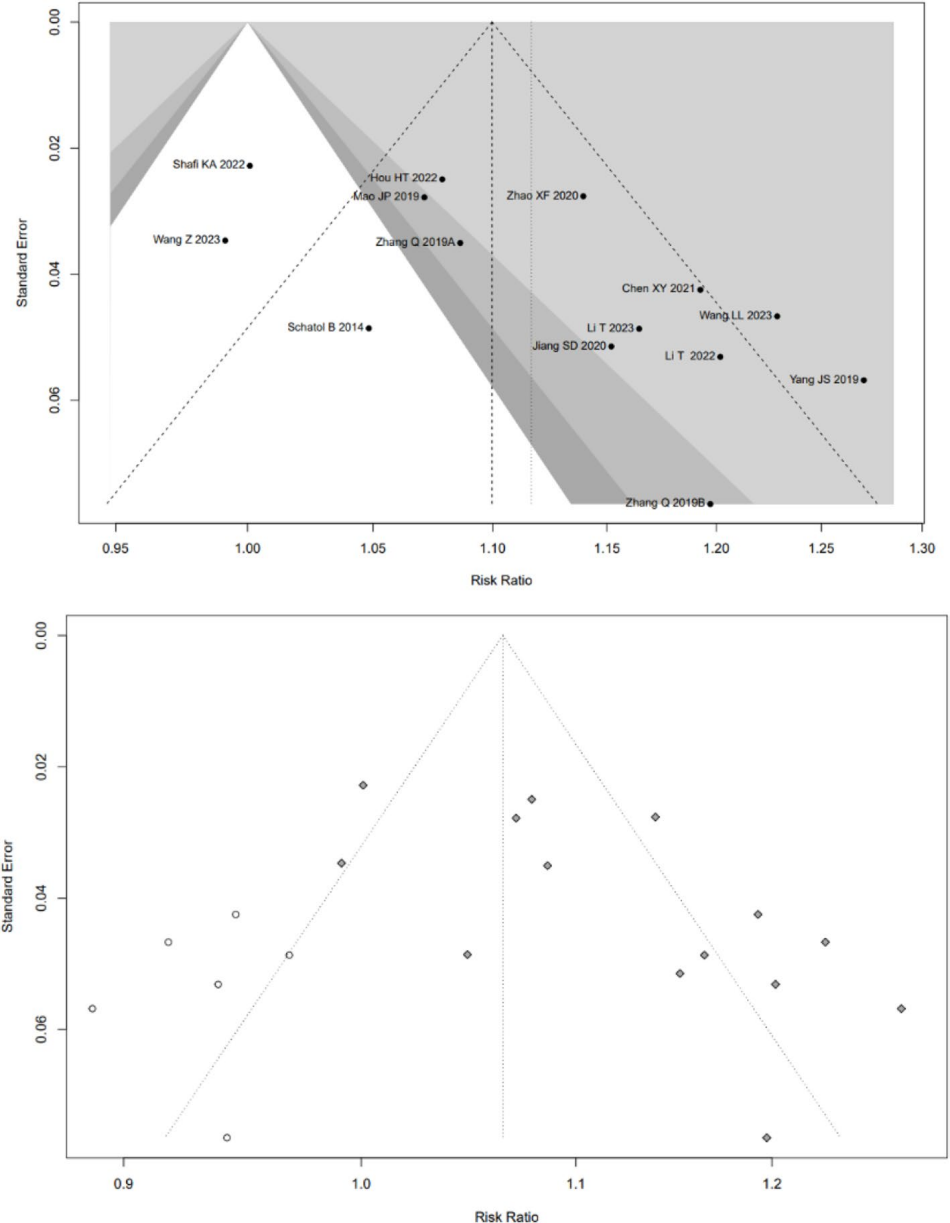
Funnel plot of CSs comparing the “perfect” accuracy of pedicle screw placement between RA and FG TLIF (top). And the shape of funnel plot after trim-and-fill method (bottom)

Low-quality evidence from fourteen CSs [20–26, 28, 31, 32, 34–37] (Table 3), reported a significant difference in “perfect” accuracy between RA and FG TLIF. RA exhibited 1.12-folds higher “perfect” accuracy than FG group, with high evidence of heterogeneity (RR: 1.1167, 95% CI: 1.0726–1.1626, z = 5.37, I^2^ = 75%, *p* < 0.0001, Fig. 4). The funnel plot demonstrates a mostly symmetrical distribution, and minimal changes are observed after applying the trim-and-fill method. This indicates a high level of confidence in the result (Fig. 5).

Subgroup analysis based on surgical type showed that RA had higher “perfect acceptable” accuracy than FG in both TLIF (RR: 1.08, 95% CI: 1.04–1.12, I^2^ = 0%, *p* < 0.05, Fig. 3a) and MIS-TLIF (RR: 1.13, 95% CI: 1.07–1.19, I^2^ = 82%, *p* < 0.05, Fig. 3a). Subgroup analysis based on robotic type indicated that Renaissance™ system, TiRobot and ROSA ™ system assisted TLIF have higher “perfect acceptable” accuracy than FG-TLIF (Fig. 3b).

A RCT reported the accuracy of pedicle screw placement with the following result [28]. Among the 92 pedicle screws in the RA group, 87 were Grade A, and 5 were Grade B. Among the 100 pedicle screws in the FG group, 85 were Grade A, and 15 were Grade B. The superiority of Grade A screws was observed in the robot-assisted MIS-TLIF group.

#### Proximal facet joint violation

**Fig. 6:**
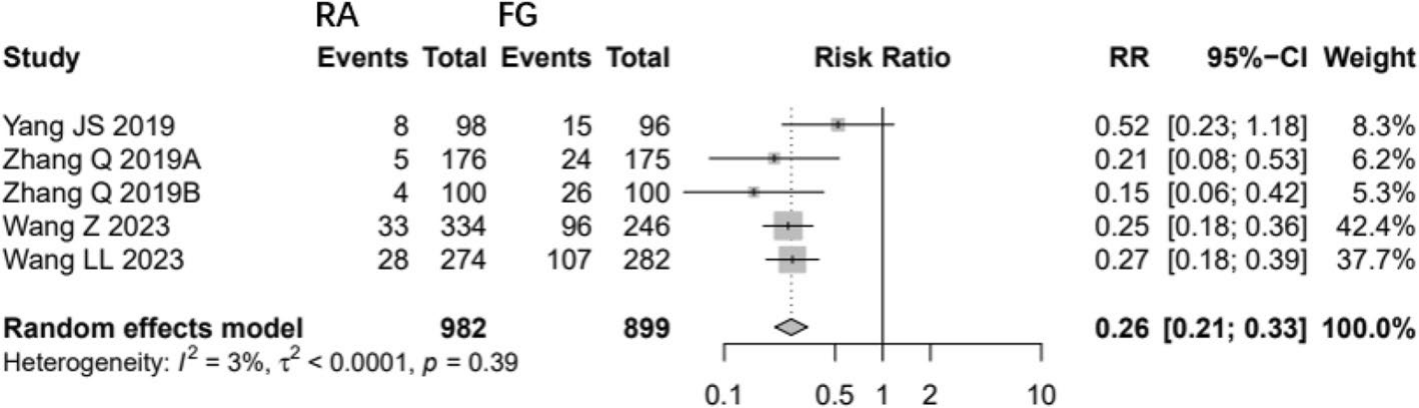
Pooled Analysis of Proximal Facet Joint Violation

Low-quality evidence from five CSs [21–23, 35, 36], reported proximal FJV assessed through CT scans. The results suggest that the patients who underwent RA pedicle screw placement had 74% fewer proximal FJV than the FG group (RR: 0.2606, 95%CI: 0.2063- 0.3293, z = -11.27, I^2^ = 3%, *p* < 0.0001) (Fig. 6).

### Secondary outcome

#### Duration of surgery

**Fig. 7:**
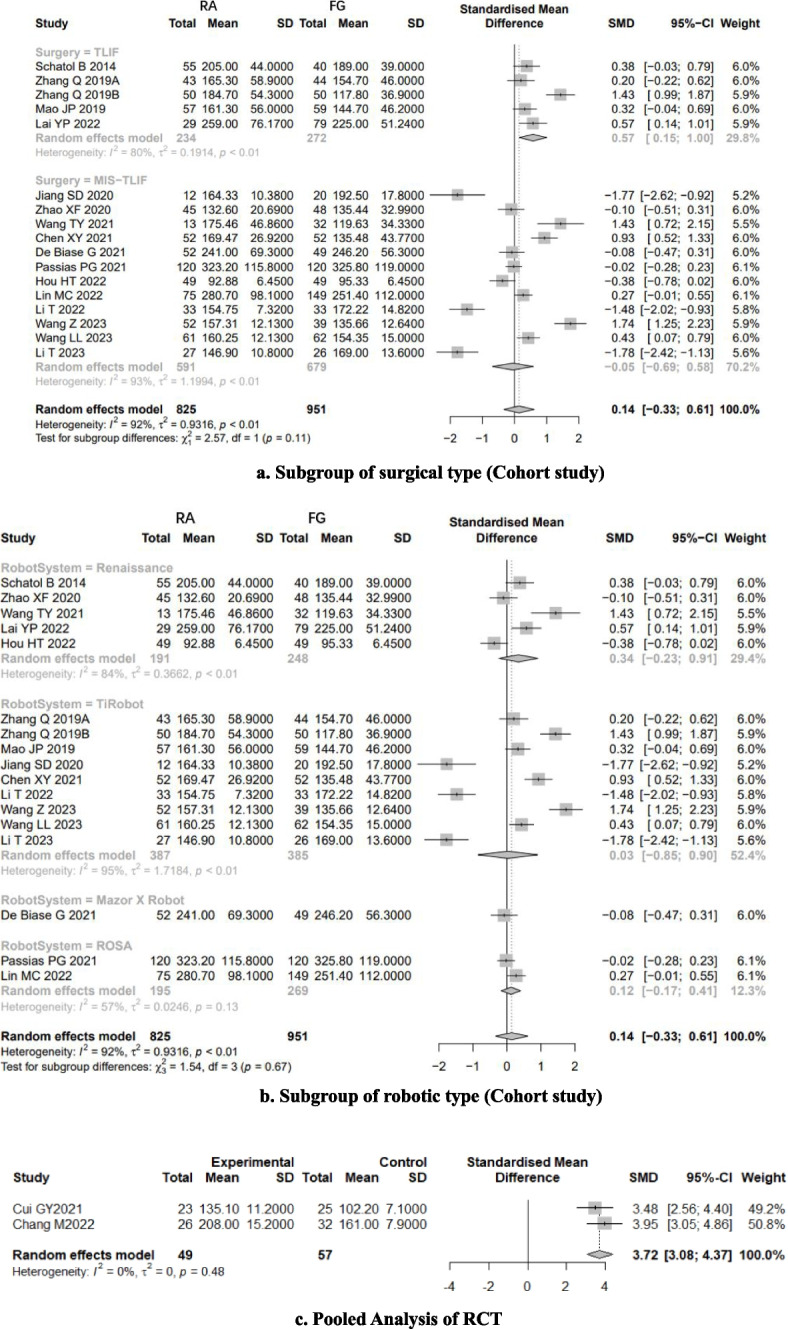
Pooled Analysis of Duration of Surgery. **a** Subgroup of surgical type (Cohort study). **b** Subgroup of robotic type (Cohort study). **c** Pooled Analysis of RCT

**Fig. 8:**
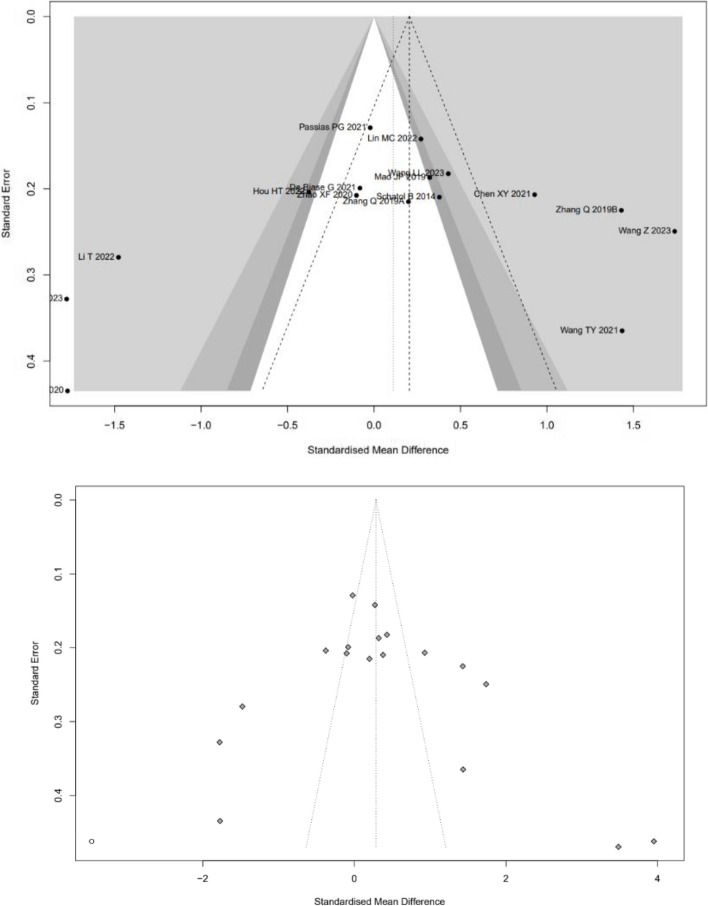
Funnel plot of CSs comparing the duration of surgery between RA-and FG-TLIF (top). And the shape of funnel plot after trim-and-fill method (bottom). No funnel plot of RCTs has been included as there were fewer than 10 RCTs

Very low-quality evidence from seventeen CSs [8, 20, 22–30, 32–37] and two RCTs [18, 19] (Table 3), reported the duration of time, as shown in Fig. 7. The results of CSs suggest that there is no significant difference between RA and FG group, with high evidence of heterogeneity (SMD: 0.1111, 95%CI: -0.391–0.6131, z = 0.43, I^2^ = 93%, *p* = 0.6646). The funnel plot demonstrates a symmetrical distribution, and the funnel plot shows minimal changes after trim-and-fill method, indicating this result with a high level of confidence (Fig. 8).

Subgroup analysis of surgical type showed that the patients who underwent RA pedicle screw placement need more surgery time than FG group in TLIF surgery (Fig. 7a). However, the duration of surgery did not show a difference between RA and FG group in MIS-TILF surgery (Fig. 7a).

According to subgroup analysis of robotic types, no robotic system outperforms the FG-TLIF in terms of duration of surgery (Fig. 7b).

And the subgroup analysis of study types [18, 19] showed that the patients who underwent RA pedicle screw placement need more surgery time (3.72 × SD minutes) than FG group (SMD: 3.7213, 95%CI: 3.0756–4.3669, z = 11.30, I^2^ = 0%, *p* < 0.0001, Fig. 7c).

#### Estimated blood loss

**Fig. 9:**
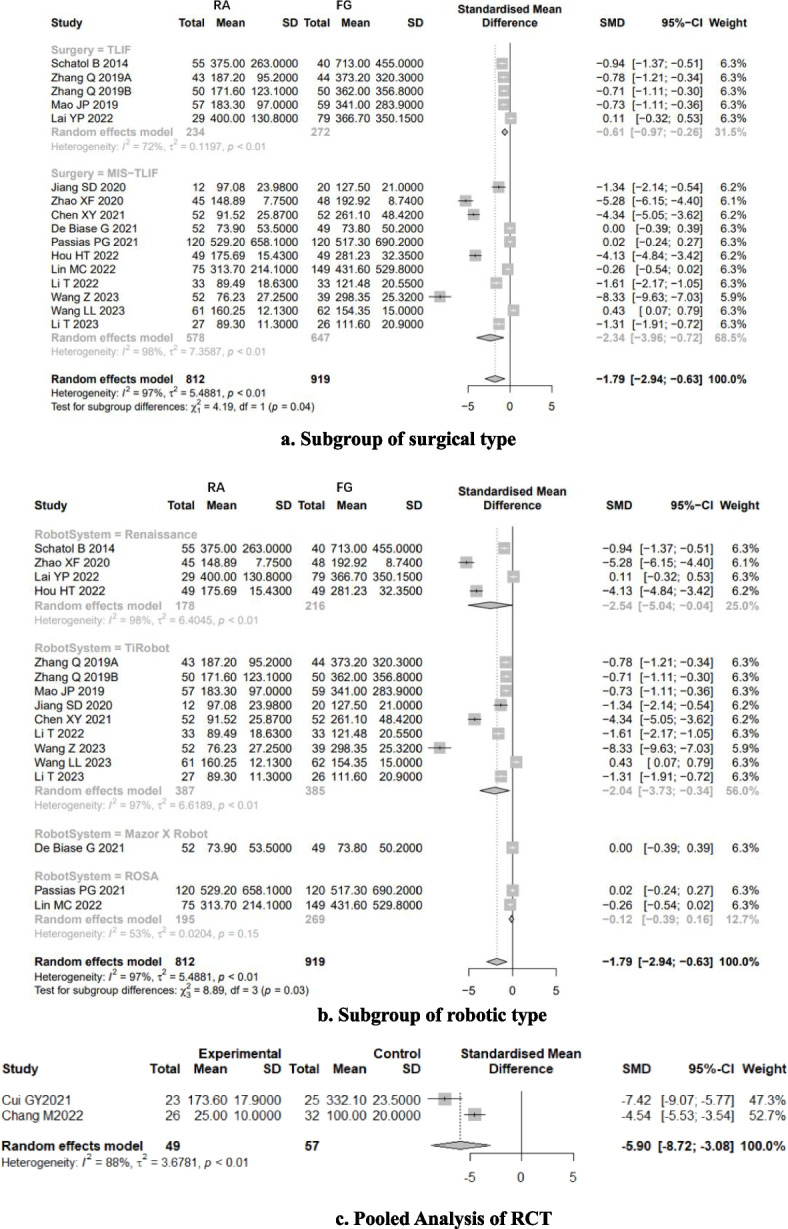
Pooled Analysis of Estimated Blood Loss. **a** Subgroup of surgical type. **b** Subgroup of robotic type. **c** Pooled Analysis of RCT

**Fig. 10:**
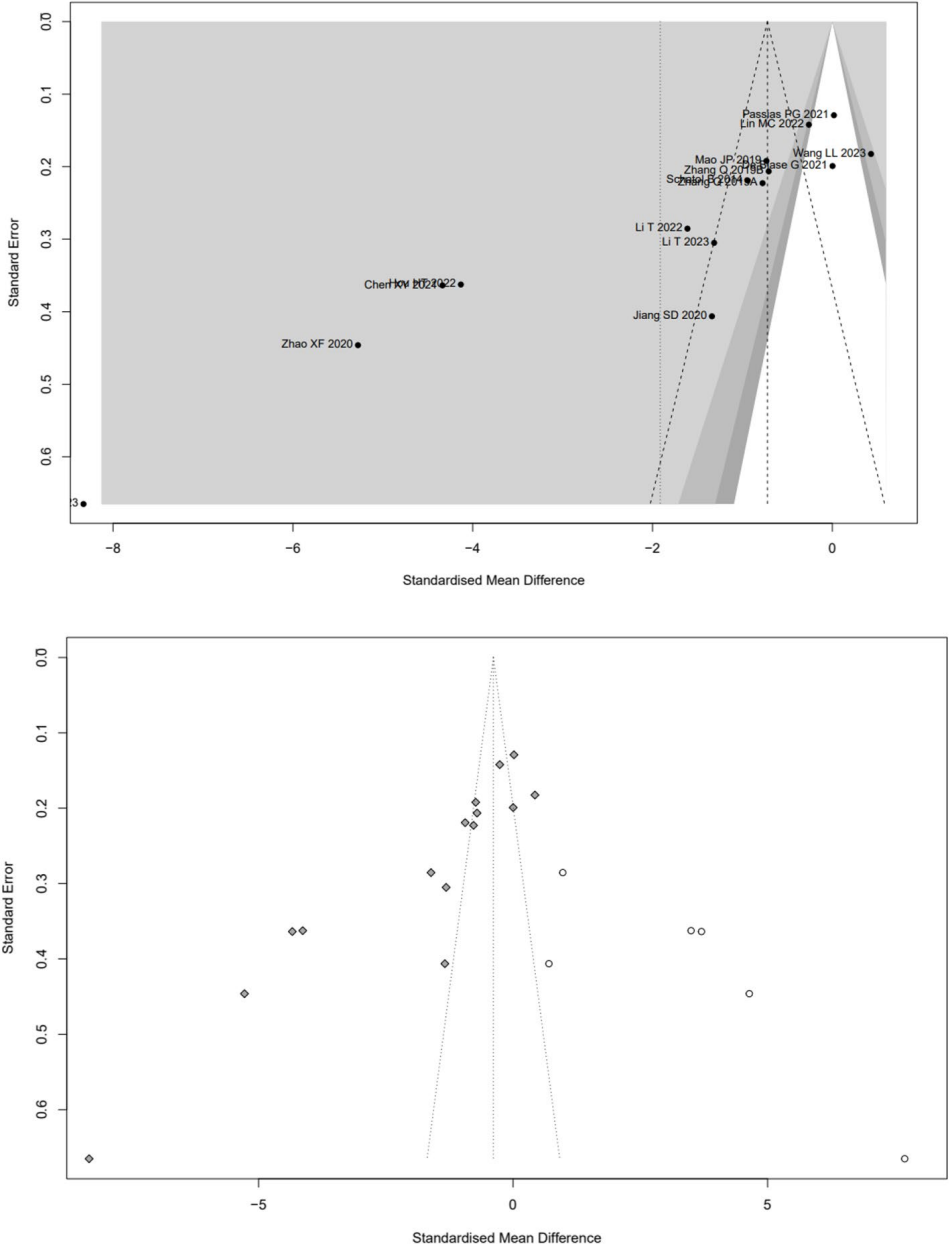
Funnel plot of CSs comparing the estimated blood loss between RA-and FG-TLIF (top). And the shape of funnel plot after trim-and-fill method (bottom). No funnel plot of RCTs has been included as there were fewer than 10 RCTs

Low-quality evidence from sixteen CSs [8, 20, 22–26, 28–30, 32–37] and two RCTs [18, 19] (Table 3), reported the estimated blood loss, as shown in Fig. 9. The results of CSs suggest that the patients who underwent RA pedicle screw placement had fewer estimated blood loss than FG group, with high evidence of heterogeneity (SMD: -1.9151, 95%CI: -3.1265–0.7036, z = -3.10, I^2^ = 98%, *p* = 0.0019). The funnel plot demonstrates a symmetrical distribution, and the funnel plot shows minimal changes after trim-and-fill method, indicating this result is reliable (Fig. 10).

Subgroup analysis of surgical type showed that the patients who underwent RA pedicle screw placement with fewer EBL than FG group both in TLIF and MIS-TLIF surgery (Fig. 9a). And Subgroup analysis of robotic type showed that the patients who underwent Renaissance™ system and TiRobot assisted pedicle screw placement with fewer EBL both in TLIF and MIS-TLIF surgery, however, the Mazor X Robot and ROSA™ do not demonstrate this advantage (Fig. 9b).

And the results of RCTs [18, 19] suggest that the patients who underwent RA pedicle screw placement had fewer estimated blood loss than FG group, with high evidence of heterogeneity (SMD: -5.9010, 95%CI: -8.7238–3.0782, z = -4.10, I^2^ = 88%, *p* < 0.0001, Fig. 9c).

#### Radiation exposure

##### Radiation time

**Fig. 11:**
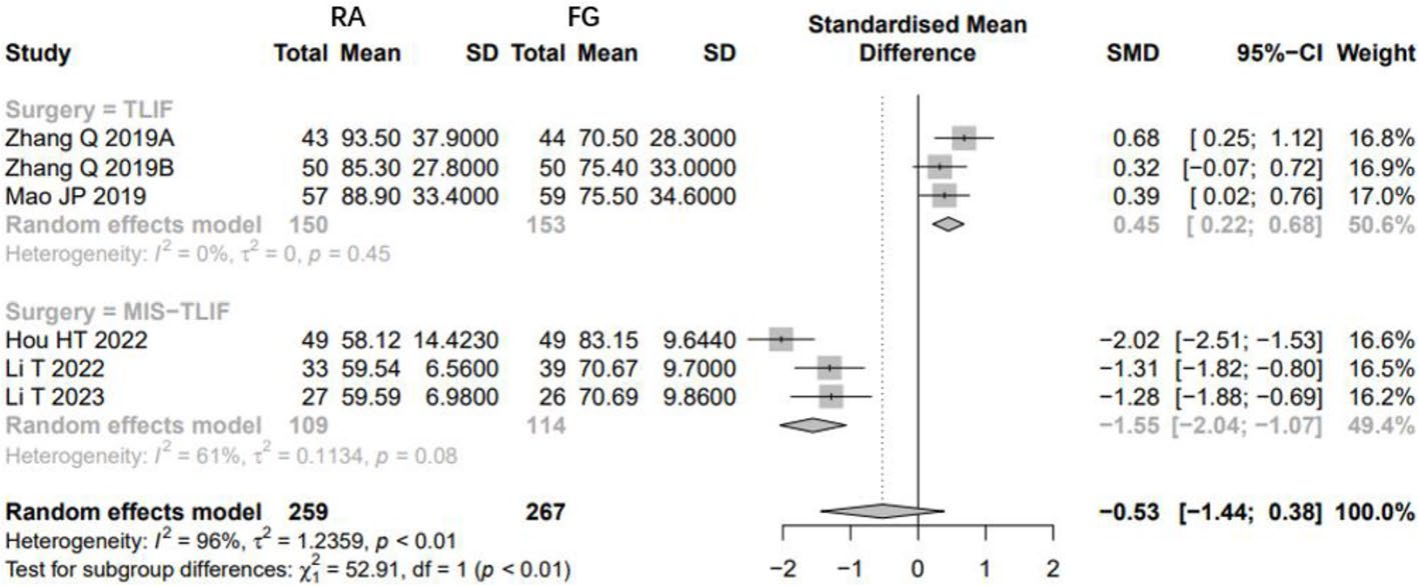
Pooled Analysis of Radiation Time

Very low-quality evidence from seven CSs [22–24, 28, 32, 34, 37] (Table 3), reported the radiation time, as shown in Fig. 11. The results of CSs suggest that there is no significant difference in radiation time between RA and FG group, with high evidence of heterogeneity (SMD: -0.5256, 95%CI: -1.4357–0.3845, z = -1.13, I^2^ = 98%, *p* = 0.2576).

Subgroup analysis of surgical type showed that the patients who underwent RA pedicle screw placement with fewer radiation exposure time in TLIF surgery, however, RA pedicle screw placement does not demonstrate this advantage when compared to FG pedicle screw placement in MIS-TLIF surgery (Fig. 11).

##### Radiation dose

**Fig. 12:**
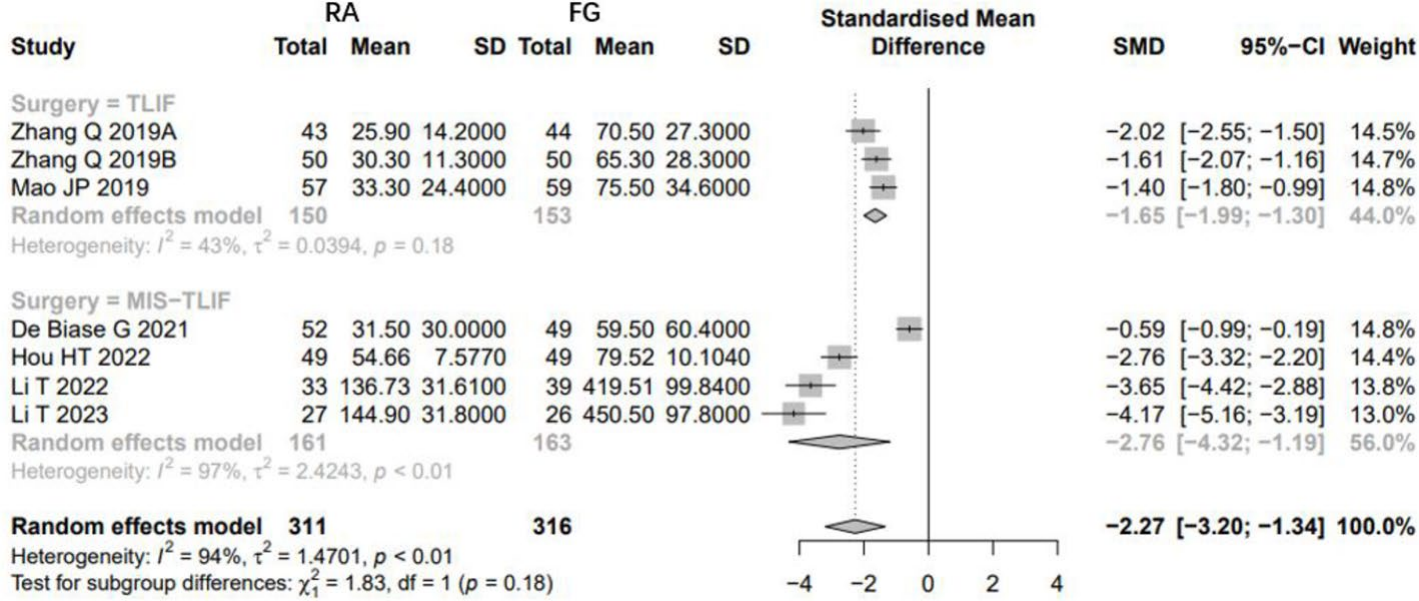
Pooled Analysis of Radiation Dose

Very low-Grade quality evidence from seven CSs [22–24, 29, 32, 34, 37] (Table 3), reported the radiation dose, as shown in Fig. 12. The results of CSs suggest that the patients who underwent RA pedicle screw placement had fewer radiation dose than FG group, with high evidence of heterogeneity (SMD: -2.2682, 95%CI: -3.1953–1.3411, z = -4.79, I^2^ = 94%, *p* < 0.0001).

Subgroup analysis of surgical type and robotic type showed that the patients who underwent RA pedicle screw placement with fewer radiation exposure dose both in TLIF and MIS-TLIF surgery.

##### Surgical revision

**Fig. 13:**
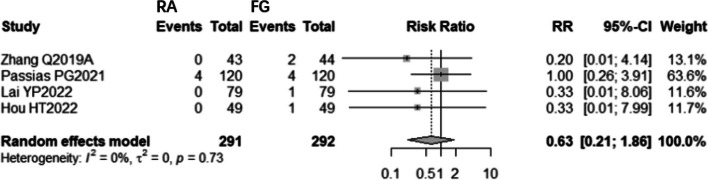
Pooled Analysis of Surgical Revision

Low-quality evidence from four CSs [8, 22, 30, 32] and one RCT [18] (Table 3), reported the number of surgical revisions, as shown in Fig. 13. The results of CSs suggest that there is no significant difference in the number of surgical revisions between RA and FG group (RR: 0.4087, 95% CI 0.1592–1.0495, z = -1.86, I^2^ = 0%, *p* = 0.0629). However, the RCT [18] reported that the number of surgical revisions of RA pedicle screw placement is lower than FG pedicle screw placement.

### Risk of bias

The Cochrane risk of bias tool was adopted evaluate the mythological quality of two RCTs, and the results were presented in Table 4. The quality of two RCTs was limited predominantly by lack of blinding, given the nature of clinical study. Regarding the random sequence generation and allocation concealment, two studies [18, 19] were low risk. In terms of blinding of outcome assessment, no information was reported to affect the outcomes because of the deviations [19]. With respect to the incomplete outcome data, two studies [18, 19] were not mentioned, thus these two studies were at unclear risks. As for selective reporting, all the RCTs were at low risk, because there is complete data and results reported with no selection. Other bias was not mentioned in these two RCTs, thus the risk of bias was unclear risks.

**Table 4:** Study quality of included RCT on the Cochrane risk-of-bias criteria

RCT	Random Sequence Generation	Allocation Concealment	Blinding of Participants and Personnel	Blinding of Outcome Assessment	Incomplete Outcome Data	Selective Reporting	Other Bias
Cui GY [18]	Low risk	Low risk	High risk	Low risk	Unclear risk	Low risk	Unclear risk
Chang M [19]	Low risk	Low risk	High risk	Unclear risk	Unclear risk	Low risk	Unclear risk

Other bias: the baseline characteristics in the experimental and control groups were different

Low quality: ether the randomization sequence generation or the allocation concealment was graded as high or unclear risk, regardless of the risk of the other items

high quality: both the randomization sequence generation or the allocation concealment was graded as low risk, and all the other items except the blinding of participants and personnel were assessed of low or unclear risk

Moderate quality: not meeting the criterion of high and low quality

The ROBINS-I was used to assess the risk of bias for four prospective cohort studies [23, 24, 26, 35] and fifteen retrospective cohort studies [8, 20–22, 25, 27–34, 36, 37] (Table 5), and detail of reasons for bias are documented in Supplemental 2.

**Table 5:** ROBINS-I (risk of bias judgements in non-randomized studies of interventions)

Author	Confounding	Selection of participants	Classification of interventions	Deviations from intended interventions	Missing data	Measurement of outcomes	Selection of reported results	Overall
Schatol B [20]	Low	Low	Low	Moderate	Low	Low	Low	Moderate
Yang JS [21]	Low	Low	Low	Low	Low	Low	Low	Low
Zhang Q [22] A	Low	Low	Low	Moderate	Low	Low	Low	Moderate
Zhang Q [23] B	Low	Moderate	Low	Moderate	Low	Low	Low	Moderate
Wang TY [27]	Low	Low	Low	Low	Low	Low	Low	Low
Chen XY [28]	Serious	Moderate	Low	Low	Low	Low	Low	Serious
Hou HT [32]	Low	Moderate	Low	Low	Low	Low	Low	Moderate
De Biase G [29]	Low	Low	Low	Low	Low	Low	Low	Low
Passias PG [8]	Low	Low	Low	Low	Low	Low	Low	Low
Lai YP [30]	Low	Moderate	Low	Low	Low	Low	Low	Moderate
Shafi KA [31]	Serious	Low	Low	Low	Low	Low	Low	Serious
Wang L [36]	Low	Moderate	Low	Moderate	Low	Low	Low	Critical
Li T [37] A	Low	Low	Low	Low	Low	Low	Low	Low
Li T [34] B	Low	Moderate	Low	Low	Low	Low	Low	Moderate
Wang Z [35]	Moderate	Low	Low	Low	Low	Low	Low	Moderate
Zhao XF [26]	Low	Moderate	Low	Low	Moderate	Low	Low	Moderate
Mao JP [24]	Low	Low	Low	Low	Low	Low	Low	Low
Jiang SD [25]	Low	Moderate	Low	Low	Low	Low	Low	Moderate
Cui GY [18]	Low	Moderate	Low	Low	Low	Low	Low	Moderate

*Low* comparable to a well-performed randomized trial, *Moderate* sound for a non-randomized study, but not comparable to a rigorous randomized trial, *Serious* presence of important problems, *Critical* too problematic to provide any useful evidence on the effects of intervention, *Overall risk of bias* equal to the most severe level of bias found in any domain

## Discussion

### Main findings and interpretation of the results

Lumbar degenerative diseases, such as spinal stenosis, disc herniation and spondylolisthesis, represent the primary causes of low back and leg pain in elderly patients [39]. When conservative treatments prove ineffective, surgical intervention becomes an inevitable option, and the choice of surgical methods varies significantly based on individual patient characteristics and their specific symptoms [40]. The conventional PLIF necessitates extensive soft tissue dissection, such as paraspinal muscles, resulting in surgical trauma and an increased risk of recurring postoperative pain [41]. This significantly impairs the postoperative quality of life for patients [42]. With the widespread promotion and application of minimally invasive techniques, there has been an increasing number of surgical options for lumbar degenerative diseases. TLIF, a technique that combines interbody fusion with pedicle screw fixation, has addressed several issues encountered in the traditional PLIF. TLIF utilizes a tube to access the intervertebral space through the intervertebral foramen, gradually expanding the muscle interval to avoid extensive soft tissue dissection. This technique effectively reduces damage to the paraspinal muscles and significantly lowers the risk of neurological and vascular injuries [43, 44]. However, the placement of channels for screw insertion and percutaneous pedicle screw fixation in TLIF requires fluoroscopic guidance, leading to extended surgical duration and increased radiation exposure compared to PLIF. In recent years, remarkable progress has been made in the clinical utilization of intelligent and digital technologies in the field of orthopedic surgery. Robot-assisted spinal surgery offers the benefits of minimally invasive procedures, enhanced precision, and reduced trauma. Through meticulous planning of optimal entry points, angles, and depths, the safety, accuracy, and precision of surgical procedures, including screw placement, have undergone significant enhancements [45, 46]. However, RA-TLIF has a steep learning curve compared to traditional FG-TLIF, requiring additional time and money. Hence, clinicians should thoroughly contemplate whether utilizing RA technology for TLIF is a more fitting choice.

The evaluation of RA pedicle screw placement primarily focuses on the accuracy of screw insertion, followed by factors such as radiation exposure, surgical duration, and blood loss. While most studies have demonstrated positive results for the RA screw placement compared to the free-hand or FG screw placement [47], there are still varying opinions in some studies. Some studies have indicated that in scenarios where anatomical structures are adequately visualized, RA screw placement may not necessarily provide a substantial accuracy advantage over traditional FG screw placement [20, 48]. Additionally, some studies indicating that RA may decrease accuracy of screw placement [49]. The debate of RA screw placement may stem from factors such as preoperative planning, image quality, and intraoperative manipulation. The automatic calculations for robot parameters still require surgeon verification, fine-tuning, or manual planning. The efficiency and accuracy of planning are closely related to image calibration and image mode selection. Currently, the automatic combination of 2D and 3D multimodal images is possible but may require more time-consuming. The design of screw placement still relies primarily on manual assessment, lacking self-planning and validation that incorporate motion and individual patient conditions. Therefore, whether RA-TLIF offers advantages in terms of accuracy, surgical time, and intraoperative blood loss over traditional FG-TLIF remains inconclusive until evidence from systematic reviews and meta-analyses becomes available.

### Screw placement accuracy

For the assessment of screw placement accuracy, the Gertzbein and Robbins criteria are commonly used [12]. Based on previous literature categorizing the accuracy of screw placement, this study considers the combination of Grade A + Grade B as "clinically acceptable" accuracy of pedicle screw placement, while Grade A is categorized as "perfect" accuracy of pedicle screw placement. We conducted a meta-analysis with pooled data from fourteen CSs [20–26, 28, 31, 32, 34–37] that included 1432 patients and 5466 cranial pedicle screws to explore whether RA-TLIF is superior to FG-TLIF in terms of “clinically acceptable” and “perfect” accuracy of pedicle screw placement. We believe that this study is the first meta-analysis to systematically compare the accuracy of pedicle screw placement between RA and FG pedicle screw placement in TLIF; however, the quality of evidence is low. The meta-analysis demonstrated that RA insertion was associated with substantially higher accuracy of pedicle screw placement than conventional FG screw insertion in TLIF. Furthermore, the pooled results of subgroup analysis suggest that RA pedicle screw placement demonstrated greater accuracy than FG in both TLIF and MIS-TLIF. In terms of robotic type, the Renaissance™ system, TiRobot, and ROSA™ system assisted TLIF have higher accuracy than FG-TLIF.

A previous study conducted by Molliqaj et al. [50] retrospectively analyzed the comparison between RA and FG screw placement in thoracolumbar fractures. The study found that RA screw placement had a higher accuracy rate compared to FG screw placement. Macke et al. [51] demonstrated the application of RA screw placement in the treatment of idiopathic scoliosis, and found a screw placement accuracy rate of 99.04% for RA placement, superior to FG placement (90.74%). Serval studies also indicate that in spinal surgeries, RA screw placement achieves significantly higher accuracy rates than FG screw placement [46, 52, 53]. However, currently, there is still a lack of evidence to suggest that RA has a superiority over traditional FG in terms of screw placement accuracy in TLIF. Generally speaking, due to the specific anatomical characteristics of each patient, RA surgery requires preoperative detailed 3D planning. Through above, the surgeon gains a comprehensive understanding of the surgical anatomical structures and reduces the likelihood of intraoperative complications. Preoperative planning also allows for optimization of implant size and trajectory based on the specific pedicle anatomy of patients. The robot system can simulate ideal screw trajectories based on individual anatomical differences and accurately reproducing these simulations during surgery. This is the primary reason why RA-TLIF.

### Proximal facet joint violation

This meta-analysis revealed that RA screw placement in TLIF can indeed reduce proximal FJV compared to FG-PLIF [21–23, 35, 36] (RR: 0.2606, 95%CI: 0.2063- 0.3293). The quality of evidence for proximal facet joint violation is low.

The accuracy of screw placement is also related to the proximal FJV [54], which has been regarded as an independent risk factor for ASD after spinal fusion [55, 56]. Sakaura et al. [57] conducted a comparative study, comparing cortical bone trajectory and traditional trajectory insertion techniques. They reported that the use of cortical bone trajectory may potentially decrease the occurrence of radiographic and systemic spinal degeneration by preserving the proximal facet joints. Levin et al. [58] pointed out that the FJV was associated with increased reoperation rates and reduced improvement in quality of life. Hyun et al. [59] conducted a prospective RCT and found no significant difference in the incidence of FJV between RA and FG insertion methods (0.00% vs. 0.71%). Similarly, Archavlis et al. [60] revealed that the occurrence of FJV in the RA group was similar to that in the FG group (5% vs. 6%). FG pedicle screw placement remains the most used technique for lumbar fusion. Meanwhile, RA screw placement has emerged as a novel minimally invasive technique, which has gradually gained acceptance for reducing screw misplacement rates and enhancing insertion safety. However, contradictory results exist regarding the incidence of FJV between FG-and RA-TLIF. We believe that the use of RA enables precise positioning, ensuring optimal screw placement within the target area of each pedicle. This minimizes the disturbance caused by pedicle screws to the adjacent proximal segment structures, reduces stress on the adjacent vertebrae, improves the biomechanical environment of the segmental structure, and ultimately decreases the probability of pseudoarthrosis and ASD.

### Perioperative indicators

According to GRADE assessment of included studies, the quality of evidence for surgery duration is very low. Surgical duration and intraoperative blood loss are perioperative indicators directly related to screw placement accuracy. Currently, there is no evidence suggesting that RA-TLIF can reduce surgical time and intraoperative blood loss. The results of this meta-analysis revealed that there was no significant difference in surgical time between the two groups [8, 20, 22–30, 32–37], and the funnel plot remained unchanged after applying the trim-and-fill method, indicating result stability. However, results of RCTs [18, 19] showed that RA had a longer surgical time compared to the FG group. Although RCTs have higher methodological quality and evidence levels than CSs, we feel that this analysis contained a greater number of moderate-quality CSs, while the number of included RCTs was limited and lacked blinding. As a result, we have greater confidence in the CS results, which show that there is no significant difference in surgical time between RA-TLIF and FG-TLIF. This may be attributed to the higher proficiency level in manual percutaneous screw placement in MIS surgery. It is speculated that as proficiency in robot usage increases, this time difference may become more prominent. Furthermore, the pooled results of subgroup analysis show that RA has a benefit over FG only in open TLIF surgery in terms of shorting surgical time, but not in MIS-TLIF surgery (SMD: 0.57, 95%CI: 0.15–1). This could be because the field of view in open TLIF surgery is greater and the operation of the surgical robot is easier, resulting in a shorter operation time than in FG-MISTLIF.

Regarding EBL, the quality of evidence for surgery duration is low. The pooled results of this study indicated that both CSs [8, 20, 22–26, 28–30, 32–37] and RCTs [18, 19] showed lower EBL with the application of RA in TLIF compared to FG. Furthermore, the surgical type and robotic type subgroups all revealed that RA screw placement accuracy can lower EBL when compared to FG screw placement accuracy. This is primarily attributed to the more accurate screw placement in RA surgeries, where the planned screw trajectory may reduce tension on the pedicle screw insertion, thus decreasing stress on the pedicle and potentially reducing tension and damage to surrounding soft tissues, such as muscles and skin.

Intraoperative radiation exposure caused by fluoroscopy is another concern to consider in TLIF [61, 62]. This study found no significant difference in radiation exposure time between the RA-TLIF and FG-TLIF [22–24, 28, 32, 34, 37], and the evidence for them are low quality. Subgroup analysis showed that RA pedicle screw placement is associated with a reduction in radiation exposure time compared to FG techniques. This suggests that the use of robotics is particularly effective in decreasing radiation exposure in open TLIF procedures. In MIS-TLIF, there is no significant difference in radiation exposure time between RA and FG techniques. This implies that, in the context of MIS-TLIF, both RA and FG may result in similar levels of radiation exposure. However, the intraoperative radiation dose in the RA group was significantly lower than in the FG group [8, 18, 19, 22, 30, 32]. And subgroup analysis has the same results.

Most studies suggest that one of the advantages of surgical robots is their ability to minimize intraoperative radiation exposure. Roser et al. [63] compared the radiation doses between RA and FG techniques and found that RA has lower doses compared to the FG group. However, Ringel et al. [49] reported no significant difference in intraoperative radiation doses between RA an FG. Schizas et al. [64] reported similar radiation times between the two groups. Based on our results of RA surgeries, there is contradictory in reducing radiation time, and the analysis indicates that the experience of the surgeon is important factors in determining radiation exposure. We believe that while RA can reduce radiation exposure in the operating room, patients often require preoperative CT scans for surgical planning, and these studies may have included the radiation time from preoperative CT scans. FG techniques rely on repeated intraoperative fluoroscopy, while RA techniques rely on the patient's preoperative 3D CT scans and preoperative planning. This is the main reason for the lack of significant difference in radiation exposure time between the two techniques.

### Surgical revision

It is important to note that the absence of a significant difference in the number of surgical revisions due to misplacement between the RA and FG screw placement in the study suggests that both techniques, when properly performed, have a similar rate of accuracy in pedicle screw placement [8, 18, 22, 30, 32]. However, the quality of evidence for surgical revision outcomes in the study is low, which affect the confidence in the results related to surgical revisions.

Surgical revision is necessary in cases of severe screw misplacement or persistent radicular pain following the initial surgery. This is because FG techniques, being the gold standard for implantation, are performed by experienced clinicians who can effectively avoid severe misplacements and postoperative complications, similar to the RA-TLIF. Nevertheless, we believe that with the advancement of modern spine surgery, the increasing complexity of spinal disorders poses higher demands on minimally invasive techniques. Robotic assistance, combined with artificial intelligence, can alleviate factors such as insufficient clinical experience, enabling more precise and accurate operations. Considering the diverse and complex clinical conditions and the need for different indications, the development of robotic technology is expected to become more refined and systematic, providing better service in the clinic.

### Limitations

Several limitations should be interpreted in this meta-analysis. The main limitation of this study is that there were too few relevant RCTs devoted to the evaluation of the difference of RA-TLIF and FG-TLIF. Thus, we did not perform the assessment of publication bias in some outcome, such as proximal facet joint violation, radiation exposure and surgical revision. Another limitation is that our study included only two RCTs, four prospective CSs and fifteen retrospective CSs. A meta-analysis of such data will lead to less powerful results compared to study obtained purely from RCTs. This difficulty primarily arises from the challenges associated with executing double-blind, randomized selection of surgical techniques in a clinical setting. Next, a limitation of this systematic review is that the general quality of the available RCTs was not high. Because studies could not blind the participants because they had the right to know about the surgery interventions., blinding of personnel and participants was impossible in practice. Investigators in most of the included studies did not describe clearly whether the outcome assessments were blinded. Moreover, our findings are limited by the heterogeneity of the included studies, therefore, the reliability of the results may be insufficient. Then, an important limitation of this study is that not all initially planned outcomes were investigated. While the primary objectives were rigorously addressed, the decision to omit certain planned outcomes introduces a potential source of bias and limits the overall comprehensiveness of our analysis. Finally, while we took rigorous measures to ensure the systematic and well-documented execution of all research procedures, data collection, and data analysis, we acknowledge that the retrospective nature of protocol registration is a serious limitation. We want to emphasize that this retrospective registration does not compromise the quality, validity, or integrity of the research findings presented in this manuscript. However, we recognize its potential impact on the perception of study transparency and pre-specification.

### Implications for future research

The results of this systematic review suggest that RA-TLIF may have certain advantages over traditional FG-TLIF. However, additional RCTs and CSs are needed to confirm these findings and provide a more comprehensive understanding of the benefits and drawbacks of each approach. Furthermore, large-scale, multicenter studies could provide more robust evidence by increasing the sample size and diversity of patient populations. Collaborative efforts can help validate the findings and enhance the generalizability of the results.

Further research in this field should focus on the following aspects. Future trials should pay attention to this area, expand the sample size, and adopt more rigorous RCT designs including the assessment of adverse effects, to incorporate additional studies in the meta-analysis. Furthermore, a critical aspect of future research should involve a comprehensive cost-effectiveness analysis comparing RA-TLIF with FG-TLIF. This would provide healthcare decision-makers with valuable information regarding the economic implications of adopting robotic technology. Last, Investigating the learning curve for surgeons adopting RA-TLIF is important. Future research should assess how surgeon experience and training impact patient outcomes to ensure safe and effective implementation of this technology.

## Conclusion

In TLIF, RA technology demonstrates more accurate placement of pedicle screws compared to FG, offering advantages in protecting adjacent facet joints and reducing intraoperative radiation dosage and blood loss. However, due to longer preoperative preparation time, the surgical duration and radiation time of RA is comparable to FG techniques. Currently, FG screw placement continues to be the predominant technique, and surgeons have greater proficiency in its application. Thus, the integration of RA into TLIF surgery may not be an optimal choice.

### Supplementary Information

Supplementary Material 1.

Supplementary Material 2.

## Data Availability

All data generated or analyzed during this study are included in this published article or are available from the corresponding author on reasonable request.
