# Supplementary material for: Comparison of robot-assisted versus fluoroscopy-guided transforaminal lumbar interbody fusion (TLIF) for lumbar degenerative diseases: a systematic review and meta-analysis of randomized controlled trails and cohort studies
Source: Syst Rev. 2024 Jul 5;13:170. doi: 10.1186/s13643-024-02600-6 (PMC11227242; doi:10.1186/s13643-024-02600-6)
Supplement: Supplementary file 1 — Supplementary Material 1. [file 13643_2024_2600_MOESM1_ESM.docx]

Supplementary material 1 - Search Strategy

Databases used:

- PubMed
- Embase (Elsevier)
- Cochrane (Wiley)
- CINAHL Database (Ebsco)
- Web of Science
- CNKI
- WanFang Database
- VIP Database

| **PubMed** |
| --- |
| 1 ‘robot surgery’ [MeSH Terms] |
| 2 ‘robot-assisted’ [Title] OR ‘robot-guided’ [Title] OR ‘robotic surgical procedures’ [Title] OR ‘robotics’ OR ‘robot-enhanced surgery’ [Title] OR ‘robotic-assisted surgeries’ [Title] OR ‘minimally invasive surgical procedures’ [Title] OR ‘MI’ OR ‘spinal fusion’ OR ‘spondylolisthesis’ OR ‘lumbar vertebrae’ OR ‘RA’ OR ‘humans’ OR ‘lumbar’ |
| 3 ‘fluoroscopy’[MeSH Terms] |
| 4 ‘fluoroscopy-guided’ [Title] OR ‘fluoroscopy’ [Title] OR ‘x-ray-guided’ [Title] OR ‘Radiography-guided’ [Title] OR ‘minimally invasive surgical procedures’ [Title] OR ‘MI’ OR ‘spinal fusion’ OR ‘spondylolisthesis’ OR ‘lumbar vertebrae’ OR ‘FG’ OR ‘humans’ OR ‘lumbar’ OR ‘barium enema’ [Title] OR ‘cineradiography’ [Title] OR ‘photofluorography’ [Title] OR ‘x-ray computed’ [Title] |
| 5 ‘spinal fusion’[MeSH Terms] |
| 6 ‘orthopedic procedures’ OR ‘arthrodesis’ OR ‘surgical procedures’ OR ‘arthrodesis’ OR ‘spondylosyndeses’ OR ‘spondylosyndesis’ |
| #7 ‘treatment outcome’ [MeSH Terms] |
| 8 ‘pedicle screws’ OR ‘blood loss’ OR ‘surgical wound infection’ OR ‘diagnosis’ OR ‘postoperative hemorrhage’ OR ‘hemorrhage’ OR ‘operative time’ OR ‘surgery time length’ OR ‘surgical time’ OR ‘length of operative time’ OR ‘facet joint’ OR ‘radiation time’ OR ‘reoperation’ OR ‘operative time’ |
| 9 ‘studies’ [MeSH Terms] |
| 10 ‘prospective studies’ OR ‘retrospective studies’ OR ‘follow-up studies’ OR ‘corhot studies’ OR ‘randomize controlled studies’ OR ‘RCT’ |
| 11 #1 OR #2 OR #3 OR #4 |
| 12 #5 OR #6 |
| 13 #7 OR #8 |
| 14 #9 OR #10 |
| 11 AND #12 AND #13 AND #14 |
| **Embase** |
| 1 ‘robot surgery’:ab,ti |
| 2 ‘robot-assisted’ OR ‘robot-guided’ OR ‘robotic surgical procedures’ OR ‘robotics’ OR ‘robot-enhanced surgery’ OR ‘robotic-assisted surgeries’ OR ‘minimally invasive surgical procedures’ OR ‘MI’ OR ‘spinal fusion’ OR ‘spondylolisthesis’ OR ‘lumbar vertebrae’ OR ‘RA’ OR ‘humans’ OR ‘lumbar’ |
| 3 ‘fluoroscopy’:ab,ti |
| 4 ‘fluoroscopy-guided’ OR ‘fluoroscopy’ OR ‘x-ray-guided’ OR ‘Radiography-guided’ OR ‘minimally invasive surgical procedures’ OR ‘MI’ OR ‘spinal fusion’ OR ‘spondylolisthesis’ OR ‘lumbar vertebrae’ OR ‘FG’ OR ‘humans’ OR ‘lumbar’ OR ‘barium enema’ OR ‘cineradiography’ OR ‘photofluorography’ OR ‘x-ray computed’ |
| 5 ‘spinal fusion’:ab,ti |
| 6 ‘orthopedic procedures’ OR ‘arthrodesis’ OR ‘surgical procedures’ OR ‘arthrodesis’ OR ‘spondylosyndeses’ OR ‘spondylosyndesis’ |
| 7 ‘treatment outcome’:ab.ti |
| 8 ‘pedicle screws’ OR ‘accuracy’ OR ‘blood loss’ OR ‘surgical wound infection’ OR ‘diagnosis’ OR ‘postoperative hemorrhage’ OR ‘hemorrhage’ OR ‘operative time’ OR ‘surgery time length’ OR ‘surgical time’ OR ‘length of operative time’ OR ‘facet joint’ OR ‘radiation time’ OR ‘reoperation’ OR ‘operative time’ OR ‘operative time’ |
| 9 #1 OR #2 OR #3 OR #4 |
| 10 #5 OR #6 |
| 11 #7 OR #8 |
| #9 AND #10 AND #11 |
| **Cochrane** |
| 1 ‘robot surgery’:ti, ab, kw |
| 2 ‘robot-assisted’ OR ‘robot-guided’ OR ‘robotic surgical procedures’ OR ‘robotics’ OR ‘robot-enhanced surgery’ OR ‘robotic-assisted surgeries’ OR ‘minimally invasive surgical procedures’ OR ‘MI’ OR ‘spinal fusion’ OR ‘spondylolisthesis’ OR ‘lumbar vertebrae’ OR ‘RA’ OR ‘humans’ OR ‘lumbar’ |
| 3 ‘fluoroscopy’:ti, ab, kw |
| 4 ‘fluoroscopy-guided’ OR ‘fluoroscopy’ OR ‘x-ray-guided’ OR ‘Radiography-guided’ OR ‘minimally invasive surgical procedures’ OR ‘MI’ OR ‘spinal fusion’ OR ‘spondylolisthesis’ OR ‘lumbar vertebrae’ OR ‘FG’ OR ‘humans’ OR ‘lumbar’ OR ‘barium enema’ OR ‘cineradiography’ OR ‘photofluorography’ OR ‘x-ray computed’ |
| 5 ‘spinal fusion’ OR ‘orthopedic procedures’ OR ‘arthrodesis’ OR ‘surgical procedures’ OR ‘arthrodesis’ OR ‘spondylosyndeses’ OR ‘spondylosyndesis’: ti,ab,kw |
| 6 ‘treatment outcome’ :ti,ab,kw |
| 7 ‘pedicle screws’ OR ‘accuracy’ OR ‘blood loss’ OR ‘surgical wound infection’ OR ‘diagnosis’ OR ‘postoperative hemorrhage’ OR ‘hemorrhage’ OR ‘operative time’ OR ‘surgery time length’ OR ‘surgical time’ OR ‘length of operative time’ OR ‘facet joint’ OR ‘radiation time’ OR ‘reoperation’ OR ‘operative time’ |
| 8 #1 OR #2 OR #3 OR #4 |
| 9 #5 OR #6 OR #7 |
| #8 AND #9 |
| **CINAHL Database (Ebsco)** |
| http://search.ebscohost.com/login.aspx?direct=true&db=rzh&bquery=((RA+%26quot%3bRandomized+Controlled+Trials%2b%26quot%3b)+OR+( RA+%26quot%3bClinical+Trials%2b%26quot%3b))+AND+((((RA+%26quot%3bFusion+MI%2c+Lumbar+1%26quot%3b)+OR+(FG+%26quot%3bFusion+MI%2c+Lumbar+2%26quot%3b)+OR+(FG+%26quot%3bFusion+MI%26quot%3b))+OR+((Robotic*)+OR+(Spinal)+OR+Lumbar)+OR+(Robot*)+OR+(Fluoroscopy*)+OR+(Robotics*+Fusion*)))+MI+(((RA+%26quot%3bSpondylolisthesis%26quot%3b))+OR+((FG+%26quot%3bSpondylolisthesis+Interviewing%26quot%3b))+OR+((RA+%26quot%3bBlood+Loss%26quot%3b))+OR+(hemorrhage*)+OR+((FG+%26quot%3bBlood+Loss%26quot%3b)+OR+(RA+%26quot%3bPostoperative+Hemorrhage%26quot%3b))+OR+reoperation+OR+pediclescrews*+OR+(pedicle+screws)+OR+surgerytime+OR+(surgery+time)+OR+((FG+%26quot%3bAccuracy%26quot%3b))))&type=1&searchMode=Standard&site=ehost-live |
| **Web of Science** |
| 1 TS = (robotics* OR robot$assisted* OR ‘robot$guided*’ OR ‘robotic$surgical$procedures*’ OR ‘robotics’ OR ‘robot$enhanced$surgery*’ OR ‘robotic$assisted$surgeries*’ OR ‘minimally$invasive$surgical$procedures*’ OR ‘MI’ OR ‘spinal$fusion*’ OR ‘spondylolisthesis’ OR ‘lumbar$vertebrae*’ OR ‘RA’ OR ‘humans’ OR ‘lumbar’ OR robotics$fusion*) |
| 2 TS = (fluoroscopy* OR fluoroscopy$guided* OR ‘X$Ray$guided*’ OR ‘Radiography$guided*’ OR ‘minimally$invasive$surgical$procedures*’ OR ‘MI’ OR ‘spinal$fusion*’ OR ‘spondylolisthesis’ OR ‘lumbar$vertebrae*’ OR ‘FG’ OR ‘humans’ OR ‘lumbar’ OR ‘barium$enema*’ OR ‘cineradiography’ OR ‘photofluorography’ OR ‘x$ray$computed*’) |
| 3 #1 AND #2 |
| 4 TS=((treatment$outcome* or ‘pedicle$screws*’ OR ‘blood$loss*’ OR ‘surgical$wound$ infection*’ OR ‘diagnosis’ OR ‘postoperative$hemorrhage*’ OR ‘hemorrhage’ OR ‘operative$time*’ OR ‘surgery$time$length*’ OR ‘surgical$time*’ OR ‘length$of$operative$time*’ OR ‘facet$joint*’ OR ‘radiation$time*’ OR ‘reoperation’ OR ‘operative$time*’) |
| 5 #3 AND #4 |
| 6 TI=(random* or blind* or placebo* or meta-analys* or trial*) OR TS=(random* or blind* or placebo* or meta-analys*) |
| #5 AND #6 |
| **CNKI** |
| （(机器人辅助+腰椎融合术+ robot-assisted+RA+lumbar fusion+微创融合+椎间孔镜融合+ minimally invasive surgical procedures+spinal fusion) OR (导航辅助+腰椎融合术+ fluoroscopy-guided+fluoroscopy +FG+lumbar fusion+微创融合+椎间孔镜融合+ minimally invasive surgical procedures+spinal fusion)） AND （围术期+术中出血量+手术时间+术后并发症+关节突关节侵犯+置钉+置钉准确性）AND （（随机对照试验+RCT）OR 临床研究 OR Trails） |
| **WanFang Database** |
| （(机器人辅助+腰椎融合术+ robot-assisted+RA+lumbar fusion+微创融合+椎间孔镜融合+ minimally invasive surgical procedures+spinal fusion) OR (导航辅助+腰椎融合术+ fluoroscopy-guided+fluoroscopy +FG+lumbar fusion+微创融合+椎间孔镜融合+ minimally invasive surgical procedures+spinal fusion)） AND （围术期+术中出血量+手术时间+术后并发症+关节突关节侵犯+置钉+置钉准确性）AND （（随机对照试验+RCT）OR 临床研究 OR Trails） |
| **VIP Database** |
| （(机器人辅助+腰椎融合术+ robot-assisted+RA+lumbar fusion+微创融合+椎间孔镜融合+ minimally invasive surgical procedures+spinal fusion) OR (导航辅助+腰椎融合术+ fluoroscopy-guided+fluoroscopy +FG+lumbar fusion+微创融合+椎间孔镜融合+ minimally invasive surgical procedures+spinal fusion)） AND （围术期+术中出血量+手术时间+术后并发症+关节突关节侵犯+置钉+置钉准确性）AND （（随机对照试验+RCT）OR 临床研究 OR Trails） |

Supplemental 2. ROBIN-I Score Consensus by Domain for Included Studies

| **Schatol B et al.** | |
| --- | --- |
| **Domain** | **Consensus** |
| **Bias Due to Confounding:**  Baseline confounding occurs when one or more prognostic variables (factors that predict the outcome of interest) also predicts the intervention received at baseline. | Low Risk. Analysis adjusted for confounders. However, the BMI was higher in the fluoroscopy-guided group, which may be potentially associated with a more demanding surgery. |
| **Bias in selection of participants into the study:**  When exclusion of some eligible participants, or the initial follow-up time of some participants, or some outcome events is related to both intervention and outcome, there will be an association between interventions and outcome even if the effects of the interventions are identical. | Low Risk. Unclear how many, if any, patients were eliminated during the pre-screening process due to comorbidities. The patients who died/withdrew consent were not included in analysis. |
| **Bias in classification of interventions:**  Bias introduced by either differential or non-differential misclassification of intervention status. Non-differential misclassification is unrelated to the outcome and will usually bias the estimated effect of intervention towards the null. Differential misclassification occurs when misclassification of intervention status is related to the outcome or the risk of the outcome and is likely to lead to bias. | Low Risk. The surgical procedure of RA-TLIF is similar to the FG-TLIF. (TLIF and decompressive). |
| **Bias due to deviations from intended interventions:**  Bias that arises when there are systematic differences between experimental intervention and comparator groups in the care provided, which represent a deviation from the intended intervention(s). Assessment of bias in this domain will depend on the type of effect of interest (either the effect of assignment to intervention or the effect of starting and adhering to intervention). | Moderate Risk. The FG-TLIF group underwent surgery using an open technique, while the larger, percutaneous robot group underwent a minimally invasive–type surgery. |
| **Bias due to missing data:**  Bias that arises when later follow-up is missing for individuals initially included and followed (such as differential loss to follow-up that is affected by prognostic factors); bias due to exclusion of individuals with missing information about intervention status or other variables such as confounders. | Low Risk. The data reported for postoperative outcomes were comprehensive. |
| **Bias in measurement of outcomes:**  Bias introduced by either differential or non-differential errors in measurement of outcome data. Such bias can arise when outcome assessors are aware of intervention status, if different methods are used to assess outcomes in different intervention groups, or if measurement errors are related to intervention status or effects. | Low Risk. Primary and second outcome was rigorously and regularly assessed by a qualified team. |
| **Bias in selection of the reported result:**  Selective reporting of results in a way that depends on the findings and prevents the estimate from being included in a meta-analysis (or other synthesis). | Low Risk. Data were reported for all patients. |
| **Overall risk of bias judgement** | Moderate Risk. No single domain was rated as having a risk of bias greater than Moderate. |

| **Yang JS et al.** | |
| --- | --- |
| **Domain** | **Consensus** |
| **Bias Due to Confounding:**  Baseline confounding occurs when one or more prognostic variables (factors that predict the outcome of interest) also predicts the intervention received at baseline. | Low Risk. Analysis adjusted for confounders, and there are no prognostic variables could predict the outcome of interest. |
| **Bias in selection of participants into the study:**  When exclusion of some eligible participants, or the initial follow-up time of some participants, or some outcome events is related to both intervention and outcome, there will be an association between interventions and outcome even if the effects of the interventions are identical. | Low Risk. “A total of 130 screws were placed under fluoroscopic guidance, with 26.2%% (34/130) penetration of the pedicle wall, and 130 screws were placed in robotic-assisted surgery, with only 6.2% (8/130) penetration of the pedicle wall.” |
| **Bias in classification of interventions:**  Bias introduced by either differential or non-differential misclassification of intervention status. Non-differential misclassification is unrelated to the outcome and will usually bias the estimated effect of intervention towards the null. Differential misclassification occurs when misclassification of intervention status is related to the outcome or the risk of the outcome and is likely to lead to bias. | Low Risk. The surgical procedure of RA-TLIF is similar to the FG-TLIF. (Minimally invasive surgery) |
| **Bias due to deviations from intended interventions:**  Bias that arises when there are systematic differences between experimental intervention and comparator groups in the care provided, which represent a deviation from the intended intervention(s). Assessment of bias in this domain will depend on the type of effect of interest (either the effect of assignment to intervention or the effect of starting and adhering to intervention). | Low Risk. All patients underwent MIS-TLIF interbody placement through a percutaneous approach with laterality determined by disease burden or clinical symptoms. Technique for interbody placement did not differ based on navigational technology, although the technique for screw placement did differ based on the utilized technology. |
| **Bias due to missing data:**  Bias that arises when later follow-up is missing for individuals initially included and followed (such as differential loss to follow-up that is affected by prognostic factors); bias due to exclusion of individuals with missing information about intervention status or other variables such as confounders. | Low Risk. No missing information about intervention status. |
| **Bias in measurement of outcomes:**  Bias introduced by either differential or non-differential errors in measurement of outcome data. Such bias can arise when outcome assessors are aware of intervention status, if different methods are used to assess outcomes in different intervention groups, or if measurement errors are related to intervention status or effects. | Low Risk. Primary and second outcome was rigorously and regularly assessed by a qualified team. |
| **Bias in selection of the reported result:**  Selective reporting of results in a way that depends on the findings and prevents the estimate from being included in a meta-analysis (or other synthesis). | Low Risk. There was no selective reporting of the results. |
| **Overall risk of bias judgement** | Low Risk. This study included domains of the same risk for bias. |

| **Zhang QA et al.** | |
| --- | --- |
| **Domain** | **Consensus** |
| **Bias Due to Confounding:**  Baseline confounding occurs when one or more prognostic variables (factors that predict the outcome of interest) also predicts the intervention received at baseline. | Low Risk. Though no confounders were reported for each group, important confounders such as age, BMI operative level were adjusted for in final analysis. “There was no statistically significant difference in age, sex, and body mass index between groups. The level and segment in patients in the RA group were similar to those in the FG group” |
| **Bias in selection of participants into the study:**  When exclusion of some eligible participants, or the initial follow-up time of some participants, or some outcome events is related to both intervention and outcome, there will be an association between interventions and outcome even if the effects of the interventions are identical. | Low Risk: A broad variety of patients were included, with no potentially biasing screening steps. |
| **Bias in classification of interventions:**  Bias introduced by either differential or non-differential misclassification of intervention status. Non-differential misclassification is unrelated to the outcome and will usually bias the estimated effect of intervention towards the null. Differential misclassification occurs when misclassification of intervention status is related to the outcome or the risk of the outcome and is likely to lead to bias. | Low Risk. The surgical procedure of RA-TLIF is similar to the FG-TLIF. (TLIF and decompressive). |
| **Bias due to deviations from intended interventions:**  Bias that arises when there are systematic differences between experimental intervention and comparator groups in the care provided, which represent a deviation from the intended intervention(s). Assessment of bias in this domain will depend on the type of effect of interest (either the effect of assignment to intervention or the effect of starting and adhering to intervention). | Moderate Risk. The FG-TLIF group underwent surgery using an open technique, while the larger, percutaneous robot group underwent a minimally invasive–type surgery. |
| **Bias due to missing data:**  Bias that arises when later follow-up is missing for individuals initially included and followed (such as differential loss to follow-up that is affected by prognostic factors); bias due to exclusion of individuals with missing information about intervention status or other variables such as confounders. | Low Risk. The data reported for postoperative primary, and second outcome were comprehensive. |
| **Bias in measurement of outcomes:**  Bias introduced by either differential or non-differential errors in measurement of outcome data. Such bias can arise when outcome assessors are aware of intervention status, if different methods are used to assess outcomes in different intervention groups, or if measurement errors are related to intervention status or effects. | Low Risk. All outcomes were rigorously and regularly assessed by a qualified team. |
| **Bias in selection of the reported result:**  Selective reporting of results in a way that depends on the findings and prevents the estimate from being included in a meta-analysis (or other synthesis). | Low Risk. Data were reported for all patients. |
| **Overall risk of bias judgement** | Moderate Risk. No single domain was rated as having a risk of bias greater than Moderate. |

| **Zhang QB et al.** | |
| --- | --- |
| **Domain** | **Consensus** |
| **Bias Due to Confounding:**  Baseline confounding occurs when one or more prognostic variables (factors that predict the outcome of interest) also predicts the intervention received at baseline. | Low Risk. “There was no statistically significant difference in age, gender, and body mass index between groups. The distribution of the cranial screw level was similar between the groups” |
| **Bias in selection of participants into the study:**  When exclusion of some eligible participants, or the initial follow-up time of some participants, or some outcome events is related to both intervention and outcome, there will be an association between interventions and outcome even if the effects of the interventions are identical. | Moderate Risk: “Patients were selected using the following inclusion criteria: (i) lumbar degenerative disease (L1–S1) resulting in radiculopathy; (ii) ineffective results with conservative treatment for no  less than 6 months.” |
| **Bias in classification of interventions:**  Bias introduced by either differential or non-differential misclassification of intervention status. Non-differential misclassification is unrelated to the outcome and will usually bias the estimated effect of intervention towards the null. Differential misclassification occurs when misclassification of intervention status is related to the outcome or the risk of the outcome and is likely to lead to bias. | Low Risk. “Robot-assisted (RA) or fluoroscopy-guided (FG) treatment was chosen by patients, after the details of these two methods were explained by the surgeon. All TLIF procedures were performed by two surgeons, who were blinded to the purpose of this study.” |
| **Bias due to deviations from intended interventions:**  Bias that arises when there are systematic differences between experimental intervention and comparator groups in the care provided, which represent a deviation from the intended intervention(s). Assessment of bias in this domain will depend on the type of effect of interest (either the effect of assignment to intervention or the effect of starting and adhering to intervention). | Moderate Risk. The FG-TLIF group underwent surgery using an open technique, while the larger, percutaneous robot group underwent a minimally invasive–type surgery |
| **Bias due to missing data:**  Bias that arises when later follow-up is missing for individuals initially included and followed (such as differential loss to follow-up that is affected by prognostic factors); bias due to exclusion of individuals with missing information about intervention status or other variables such as confounders. | Low Risk. The data reported for postoperative outcomes were comprehensive. |
| **Bias in measurement of outcomes:**  Bias introduced by either differential or non-differential errors in measurement of outcome data. Such bias can arise when outcome assessors are aware of intervention status, if different methods are used to assess outcomes in different intervention groups, or if measurement errors are related to intervention status or effects. | Low Risk. Primary and second outcome was rigorously and regularly assessed by a qualified team. |
| **Bias in selection of the reported result:**  Selective reporting of results in a way that depends on the findings and prevents the estimate from being included in a meta-analysis (or other synthesis). | Low Risk. Data were reported for all patients. |
| **Overall risk of bias judgement** | Moderate Risk. No single domain was rated as having a risk of bias greater than Moderate. |

| **Wang TY et al.** | |
| --- | --- |
| **Domain** | **Consensus** |
| **Bias Due to Confounding:**  Baseline confounding occurs when one or more prognostic variables (factors that predict the outcome of interest) also predicts the intervention received at baseline. | Low Risk. Patients with or without laminectomy, instrumentation removal, or extension to previous fusion were also included if they also underwent concurrent MIS-TLIF. No confounders were reported for each group. |
| **Bias in selection of participants into the study:**  When exclusion of some eligible participants, or the initial follow-up time of some participants, or some outcome events is related to both intervention and outcome, there will be an association between interventions and outcome even if the effects of the interventions are identical. | No information. The N value was so low as to have very little statistical meaning and also the selection process is difficult to discern. |
| **Bias in classification of interventions:**  Bias introduced by either differential or non-differential misclassification of intervention status. Non-differential misclassification is unrelated to the outcome and will usually bias the estimated effect of intervention towards the null. Differential misclassification occurs when misclassification of intervention status is related to the outcome or the risk of the outcome and is likely to lead to bias. | Low Risk. The surgical procedure of RA-MISTLIF is similar to the FG-MISTLIF. |
| **Bias due to deviations from intended interventions:**  Bias that arises when there are systematic differences between experimental intervention and comparator groups in the care provided, which represent a deviation from the intended intervention(s). Assessment of bias in this domain will depend on the type of effect of interest (either the effect of assignment to intervention or the effect of starting and adhering to intervention). | Low Risk. The MIS-TLIF was applied properly and with consistency. |
| **Bias due to missing data:**  Bias that arises when later follow-up is missing for individuals initially included and followed (such as differential loss to follow-up that is affected by prognostic factors); bias due to exclusion of individuals with missing information about intervention status or other variables such as confounders. | Low Risk. The data reported for postoperative primary, and second outcome were comprehensive. |
| **Bias in measurement of outcomes:**  Bias introduced by either differential or non-differential errors in measurement of outcome data. Such bias can arise when outcome assessors are aware of intervention status, if different methods are used to assess outcomes in different intervention groups, or if measurement errors are related to intervention status or effects. | Low Risk A trained research assistant conducted structured interviews preoperatively. All cases were validated by a second investigator. |
| **Bias in selection of the reported result:**  Selective reporting of results in a way that depends on the findings and prevents the estimate from being included in a meta-analysis (or other synthesis). | Low Risk. Data were reported for all patients. |
| **Overall risk of bias judgement** | Low Risk. This study included domains of the same risk for bias. |

| **Chen XY et al.** | |
| --- | --- |
| **Domain** | **Consensus** |
| **Bias Due to Confounding:**  Baseline confounding occurs when one or more prognostic variables (factors that predict the outcome of interest) also predicts the intervention received at baseline. | Serious Risk. No adjustment, univariate analysis only. |
| **Bias in selection of participants into the study:**  When exclusion of some eligible participants, or the initial follow-up time of some participants, or some outcome events is related to both intervention and outcome, there will be an association between interventions and outcome even if the effects of the interventions are identical. | Moderate Risk. Patients from this study were selected from a much larger study, and only 104 patients from the parent study were included. |
| **Bias in classification of interventions:**  Bias introduced by either differential or non-differential misclassification of intervention status. Non-differential misclassification is unrelated to the outcome and will usually bias the estimated effect of intervention towards the null. Differential misclassification occurs when misclassification of intervention status is related to the outcome or the risk of the outcome and is likely to lead to bias. | Low Risk. The surgical procedure of RA-TLIF is similar to the FG-TLIF. |
| **Bias due to deviations from intended interventions:**  Bias that arises when there are systematic differences between experimental intervention and comparator groups in the care provided, which represent a deviation from the intended intervention(s). Assessment of bias in this domain will depend on the type of effect of interest (either the effect of assignment to intervention or the effect of starting and adhering to intervention). | Low Risk: There were no systematic processes affecting the designation of RA-TLIF versus FG-TLIF. |
| **Bias due to missing data:**  Bias that arises when later follow-up is missing for individuals initially included and followed (such as differential loss to follow-up that is affected by prognostic factors); bias due to exclusion of individuals with missing information about intervention status or other variables such as confounders. | Low Risk. The data reported for postoperative primary, and second outcome were comprehensive. |
| **Bias in measurement of outcomes:**  Bias introduced by either differential or non-differential errors in measurement of outcome data. Such bias can arise when outcome assessors are aware of intervention status, if different methods are used to assess outcomes in different intervention groups, or if measurement errors are related to intervention status or effects. | Low Risk. Primary and second outcome was rigorously and regularly assessed by a qualified team. |
| **Bias in selection of the reported result:**  Selective reporting of results in a way that depends on the findings and prevents the estimate from being included in a meta-analysis (or other synthesis). | Low Risk. Data were reported for all patients. |
| **Overall risk of bias judgement** | Serious Risk. Due to confounding |

| **Hou HT et al.** | |
| --- | --- |
| **Domain** | **Consensus** |
| **Bias Due to Confounding:**  Baseline confounding occurs when one or more prognostic variables (factors that predict the outcome of interest) also predicts the intervention received at baseline. | Low Risk. No confounders were reported for each group. No significant differences were found between these two groups in terms of age, sex, BMI, primary diagnosis, operated level and ASA grade |
| **Bias in selection of participants into the study:**  When exclusion of some eligible participants, or the initial follow-up time of some participants, or some outcome events is related to both intervention and outcome, there will be an association between interventions and outcome even if the effects of the interventions are identical. | Moderate Risk. 15 of patients refused consent, and 5 patients dropped out after enrollment. |
| **Bias in classification of interventions:**  Bias introduced by either differential or non-differential misclassification of intervention status. Non-differential misclassification is unrelated to the outcome and will usually bias the estimated effect of intervention towards the null. Differential misclassification occurs when misclassification of intervention status is related to the outcome or the risk of the outcome and is likely to lead to bias. | Low Risk. The surgical procedure of RA-MISTLIF is similar to the FG-MISTLIF. |
| **Bias due to deviations from intended interventions:**  Bias that arises when there are systematic differences between experimental intervention and comparator groups in the care provided, which represent a deviation from the intended intervention(s). Assessment of bias in this domain will depend on the type of effect of interest (either the effect of assignment to intervention or the effect of starting and adhering to intervention). | Low Risk. The MIS-TLIF was applied properly and with consistency. |
| **Bias due to missing data:**  Bias that arises when later follow-up is missing for individuals initially included and followed (such as differential loss to follow-up that is affected by prognostic factors); bias due to exclusion of individuals with missing information about intervention status or other variables such as confounders. | Low Risk. The data reported for postoperative primary, and second outcome were comprehensive. |
| **Bias in measurement of outcomes:**  Bias introduced by either differential or non-differential errors in measurement of outcome data. Such bias can arise when outcome assessors are aware of intervention status, if different methods are used to assess outcomes in different intervention groups, or if measurement errors are related to intervention status or effects. | Low Risk. Primary and second outcome was rigorously and regularly assessed by a qualified team. |
| **Bias in selection of the reported result:**  Selective reporting of results in a way that depends on the findings and prevents the estimate from being included in a meta-analysis (or other synthesis). | Low Risk. Data were reported for all patients. |
| **Overall risk of bias judgement** | Moderate Risk. No single domain was rated as having a risk of bias greater than Moderate. |

| **De Biase G et al.** | |
| --- | --- |
| **Domain** | **Consensus** |
| **Bias Due to Confounding:**  Baseline confounding occurs when one or more prognostic variables (factors that predict the outcome of interest) also predicts the intervention received at baseline. | Low Risk. No confounders were reported for each group. |
| **Bias in selection of participants into the study:**  When exclusion of some eligible participants, or the initial follow-up time of some participants, or some outcome events is related to both intervention and outcome, there will be an association between interventions and outcome even if the effects of the interventions are identical. | Low Risk. Unclear how many, if any, patients were eliminated during the pre-screening process due to comorbidities. The patients who died/withdrew consent were not included in analysis. |
| **Bias in classification of interventions:**  Bias introduced by either differential or non-differential misclassification of intervention status. Non-differential misclassification is unrelated to the outcome and will usually bias the estimated effect of intervention towards the null. Differential misclassification occurs when misclassification of intervention status is related to the outcome or the risk of the outcome and is likely to lead to bias. | Low Risk. The surgical procedure of RA-MISTLIF is similar to the FG-MISTLIF. |
| **Bias due to deviations from intended interventions:**  Bias that arises when there are systematic differences between experimental intervention and comparator groups in the care provided, which represent a deviation from the intended intervention(s). Assessment of bias in this domain will depend on the type of effect of interest (either the effect of assignment to intervention or the effect of starting and adhering to intervention). | Low Risk. The MIS-TLIF was applied properly and with consistency. |
| **Bias due to missing data:**  Bias that arises when later follow-up is missing for individuals initially included and followed (such as differential loss to follow-up that is affected by prognostic factors); bias due to exclusion of individuals with missing information about intervention status or other variables such as confounders. | Low Risk. The data reported for postoperative primary, and second outcome were comprehensive. |
| **Bias in measurement of outcomes:**  Bias introduced by either differential or non-differential errors in measurement of outcome data. Such bias can arise when outcome assessors are aware of intervention status, if different methods are used to assess outcomes in different intervention groups, or if measurement errors are related to intervention status or effects. | Low Risk. A trained research assistant conducted structured interviews preoperatively. All cases were validated by a second investigator. |
| **Bias in selection of the reported result:**  Selective reporting of results in a way that depends on the findings and prevents the estimate from being included in a meta-analysis (or other synthesis). | Low Risk. Data were reported for all patients. |
| **Overall risk of bias judgement** | Low Risk. This study included domains of the same risk for bias. |

| **Passias PG et al.** | |
| --- | --- |
| **Domain** | **Consensus** |
| **Bias Due to Confounding:**  Baseline confounding occurs when one or more prognostic variables (factors that predict the outcome of interest) also predicts the intervention received at baseline. | Low Risk. No confounders were reported for each group. |
| **Bias in selection of participants into the study:**  When exclusion of some eligible participants, or the initial follow-up time of some participants, or some outcome events is related to both intervention and outcome, there will be an association between interventions and outcome even if the effects of the interventions are identical. | Low Risk. A broad variety of patients were included, with no potentially biasing screening steps. |
| **Bias in classification of interventions:**  Bias introduced by either differential or non-differential misclassification of intervention status. Non-differential misclassification is unrelated to the outcome and will usually bias the estimated effect of intervention towards the null. Differential misclassification occurs when misclassification of intervention status is related to the outcome or the risk of the outcome and is likely to lead to bias. | Low Risk. The surgical procedure of RA-MISTLIF is similar to the FG-MISTLIF. |
| **Bias due to deviations from intended interventions:**  Bias that arises when there are systematic differences between experimental intervention and comparator groups in the care provided, which represent a deviation from the intended intervention(s). Assessment of bias in this domain will depend on the type of effect of interest (either the effect of assignment to intervention or the effect of starting and adhering to intervention). | Low Risk. The MIS-TLIF was applied properly and with consistency. |
| **Bias due to missing data:**  Bias that arises when later follow-up is missing for individuals initially included and followed (such as differential loss to follow-up that is affected by prognostic factors); bias due to exclusion of individuals with missing information about intervention status or other variables such as confounders. | Low Risk. The data reported for postoperative primary, and second outcome were comprehensive. |
| **Bias in measurement of outcomes:**  Bias introduced by either differential or non-differential errors in measurement of outcome data. Such bias can arise when outcome assessors are aware of intervention status, if different methods are used to assess outcomes in different intervention groups, or if measurement errors are related to intervention status or effects. | Low Risk. Primary and second outcome was rigorously and regularly assessed by a qualified team. |
| **Bias in selection of the reported result:**  Selective reporting of results in a way that depends on the findings and prevents the estimate from being included in a meta-analysis (or other synthesis). | Low Risk. Data were reported for all patients. |
| **Overall risk of bias judgement** | Low Risk. This study included domains of the same risk for bias. |

| **Lai YP et al.** | |
| --- | --- |
| **Domain** | **Consensus** |
| **Bias Due to Confounding:**  Baseline confounding occurs when one or more prognostic variables (factors that predict the outcome of interest) also predicts the intervention received at baseline. | Low Risk. No confounders were reported for each group. |
| **Bias in selection of participants into the study:**  When exclusion of some eligible participants, or the initial follow-up time of some participants, or some outcome events is related to both intervention and outcome, there will be an association between interventions and outcome even if the effects of the interventions are identical. | Low Risk. A total of 185 consecutive patients underwent TLIF, and twenty-nine cases of revision and one case of infection were excluded. Finally, 29 patients were assigned to the RA TLIF group, and 79 patients were assigned to the FG TLIF group |
| **Bias in classification of interventions:**  Bias introduced by either differential or non-differential misclassification of intervention status. Non-differential misclassification is unrelated to the outcome and will usually bias the estimated effect of intervention towards the null. Differential misclassification occurs when misclassification of intervention status is related to the outcome or the risk of the outcome and is likely to lead to bias. | Low Risk. The surgical procedure of RA-TLIF is similar to the FG-TLIF. |
| **Bias due to deviations from intended interventions:**  Bias that arises when there are systematic differences between experimental intervention and comparator groups in the care provided, which represent a deviation from the intended intervention(s). Assessment of bias in this domain will depend on the type of effect of interest (either the effect of assignment to intervention or the effect of starting and adhering to intervention). | Low Risk. The TLIF was applied properly and with consistency. |
| **Bias due to missing data:**  Bias that arises when later follow-up is missing for individuals initially included and followed (such as differential loss to follow-up that is affected by prognostic factors); bias due to exclusion of individuals with missing information about intervention status or other variables such as confounders. | Low Risk. The data reported for postoperative primary, and second outcome were comprehensive. |
| **Bias in measurement of outcomes:**  Bias introduced by either differential or non-differential errors in measurement of outcome data. Such bias can arise when outcome assessors are aware of intervention status, if different methods are used to assess outcomes in different intervention groups, or if measurement errors are related to intervention status or effects. | Low Risk. Primary and second outcome was rigorously and regularly assessed by a qualified team. |
| **Bias in selection of the reported result:**  Selective reporting of results in a way that depends on the findings and prevents the estimate from being included in a meta-analysis (or other synthesis). | Low Risk. Data were reported for all patients. |
| **Overall risk of bias judgement** | Moderate Risk. No single domain was rated as having a risk of bias greater than Moderate. |

| **Shafi KA et al.** | |
| --- | --- |
| **Domain** | **Consensus** |
| **Bias Due to Confounding:**  Baseline confounding occurs when one or more prognostic variables (factors that predict the outcome of interest) also predicts the intervention received at baseline. | Serious Risk. No adjustment, univariate analysis only. |
| **Bias in selection of participants into the study:**  When exclusion of some eligible participants, or the initial follow-up time of some participants, or some outcome events is related to both intervention and outcome, there will be an association between interventions and outcome even if the effects of the interventions are identical. | Low Risk. A broad variety of patients were included, with no potentially biasing screening steps. |
| **Bias in classification of interventions:**  Bias introduced by either differential or non-differential misclassification of intervention status. Non-differential misclassification is unrelated to the outcome and will usually bias the estimated effect of intervention towards the null. Differential misclassification occurs when misclassification of intervention status is related to the outcome or the risk of the outcome and is likely to lead to bias. | Low Risk. The surgical procedure of RA-MISTLIF is similar to the FG-MISTLIF. |
| **Bias due to deviations from intended interventions:**  Bias that arises when there are systematic differences between experimental intervention and comparator groups in the care provided, which represent a deviation from the intended intervention(s). Assessment of bias in this domain will depend on the type of effect of interest (either the effect of assignment to intervention or the effect of starting and adhering to intervention). | Low Risk. The MIS-TLIF was applied properly and with consistency. |
| **Bias due to missing data:**  Bias that arises when later follow-up is missing for individuals initially included and followed (such as differential loss to follow-up that is affected by prognostic factors); bias due to exclusion of individuals with missing information about intervention status or other variables such as confounders. | Low Risk. The data reported for postoperative primary, and second outcome were comprehensive. |
| **Bias in measurement of outcomes:**  Bias introduced by either differential or non-differential errors in measurement of outcome data. Such bias can arise when outcome assessors are aware of intervention status, if different methods are used to assess outcomes in different intervention groups, or if measurement errors are related to intervention status or effects. | Low Risk. The data reported for postoperative primary, and second outcome were comprehensive. |
| **Bias in selection of the reported result:**  Selective reporting of results in a way that depends on the findings and prevents the estimate from being included in a meta-analysis (or other synthesis). | Low Risk. Data were reported for all patients. |
| **Overall risk of bias judgement** | Serious Risk. Due to Confounding. |

| **Wang L et al.** | |
| --- | --- |
| **Domain** | **Consensus** |
| **Bias Due to Confounding:**  Baseline confounding occurs when one or more prognostic variables (factors that predict the outcome of interest) also predicts the intervention received at baseline. | Low Risk. No confounders were reported for each group. |
| **Bias in selection of participants into the study:**  When exclusion of some eligible participants, or the initial follow-up time of some participants, or some outcome events is related to both intervention and outcome, there will be an association between interventions and outcome even if the effects of the interventions are identical. | Moderate Risk. Only patients who underwent single-level  and double-level lumbar surgery were included in this study; longer surgical segments are needed to confirm the findings. |
| **Bias in classification of interventions:**  Bias introduced by either differential or non-differential misclassification of intervention status. Non-differential misclassification is unrelated to the outcome and will usually bias the estimated effect of intervention towards the null. Differential misclassification occurs when misclassification of intervention status is related to the outcome or the risk of the outcome and is likely to lead to bias. | Low Risk. The surgical procedure of RA-MISTLIF is similar to the FG-MISTLIF. |
| **Bias due to deviations from intended interventions:**  Bias that arises when there are systematic differences between experimental intervention and comparator groups in the care provided, which represent a deviation from the intended intervention(s). Assessment of bias in this domain will depend on the type of effect of interest (either the effect of assignment to intervention or the effect of starting and adhering to intervention). | Moderate Risk. The FG-TLIF group underwent surgery using an open technique, while the larger, percutaneous robot group underwent a minimally invasive–type surgery. |
| **Bias due to missing data:**  Bias that arises when later follow-up is missing for individuals initially included and followed (such as differential loss to follow-up that is affected by prognostic factors); bias due to exclusion of individuals with missing information about intervention status or other variables such as confounders. | Low Risk. The data reported for postoperative primary, and second outcome were comprehensive. |
| **Bias in measurement of outcomes:**  Bias introduced by either differential or non-differential errors in measurement of outcome data. Such bias can arise when outcome assessors are aware of intervention status, if different methods are used to assess outcomes in different intervention groups, or if measurement errors are related to intervention status or effects. | Low Risk. Primary and second outcome was rigorously and regularly assessed by a qualified team. |
| **Bias in selection of the reported result:**  Selective reporting of results in a way that depends on the findings and prevents the estimate from being included in a meta-analysis (or other synthesis). | Low Risk. Data were reported for all patients. |
| **Overall risk of bias judgement** | Moderate Risk. No single domain was rated as having a risk of bias greater than Moderate. |

| **Li T A et al.** | |
| --- | --- |
| **Domain** | **Consensus** |
| **Bias Due to Confounding:**  Baseline confounding occurs when one or more prognostic variables (factors that predict the outcome of interest) also predicts the intervention received at baseline. | Low Risk. No confounders were reported for each group. |
| **Bias in selection of participants into the study:**  When exclusion of some eligible participants, or the initial follow-up time of some participants, or some outcome events is related to both intervention and outcome, there will be an association between interventions and outcome even if the effects of the interventions are identical. | Low Risk. A broad variety of patients were included, with no potentially biasing screening steps. |
| **Bias in classification of interventions:**  Bias introduced by either differential or non-differential misclassification of intervention status. Non-differential misclassification is unrelated to the outcome and will usually bias the estimated effect of intervention towards the null. Differential misclassification occurs when misclassification of intervention status is related to the outcome or the risk of the outcome and is likely to lead to bias. | Low Risk. The surgical procedure of RA-MISTLIF is similar to the FG-MISTLIF. |
| **Bias due to deviations from intended interventions:**  Bias that arises when there are systematic differences between experimental intervention and comparator groups in the care provided, which represent a deviation from the intended intervention(s). Assessment of bias in this domain will depend on the type of effect of interest (either the effect of assignment to intervention or the effect of starting and adhering to intervention). | Low Risk. The MIS-TLIF was applied properly and with consistency. |
| **Bias due to missing data:**  Bias that arises when later follow-up is missing for individuals initially included and followed (such as differential loss to follow-up that is affected by prognostic factors); bias due to exclusion of individuals with missing information about intervention status or other variables such as confounders. | Low Risk. The data reported for postoperative primary, and second outcome were comprehensive. |
| **Bias in measurement of outcomes:**  Bias introduced by either differential or non-differential errors in measurement of outcome data. Such bias can arise when outcome assessors are aware of intervention status, if different methods are used to assess outcomes in different intervention groups, or if measurement errors are related to intervention status or effects. | Low Risk. The data reported for postoperative primary, and second outcome were comprehensive. |
| **Bias in selection of the reported result:**  Selective reporting of results in a way that depends on the findings and prevents the estimate from being included in a meta-analysis (or other synthesis). | Low Risk. Data were reported for all patients. |
| **Overall risk of bias judgement** | Low Risk. This study included domains of the same risk for bias. |

| **Li T B et al.** | |
| --- | --- |
| **Domain** | **Consensus** |
| **Bias Due to Confounding:**  Baseline confounding occurs when one or more prognostic variables (factors that predict the outcome of interest) also predicts the intervention received at baseline. | Low Risk. No confounders were reported for each group. |
| **Bias in selection of participants into the study:**  When exclusion of some eligible participants, or the initial follow-up time of some participants, or some outcome events is related to both intervention and outcome, there will be an association between interventions and outcome even if the effects of the interventions are identical. | Moderate Risk. Patients from this study were selected from a much larger study, and only 72 patients from the parent study were included. |
| **Bias in classification of interventions:**  Bias introduced by either differential or non-differential misclassification of intervention status. Non-differential misclassification is unrelated to the outcome and will usually bias the estimated effect of intervention towards the null. Differential misclassification occurs when misclassification of intervention status is related to the outcome or the risk of the outcome and is likely to lead to bias. | Low Risk. The surgical procedure of RA-MISTLIF is similar to the FG-MISTLIF. |
| **Bias due to deviations from intended interventions:**  Bias that arises when there are systematic differences between experimental intervention and comparator groups in the care provided, which represent a deviation from the intended intervention(s). Assessment of bias in this domain will depend on the type of effect of interest (either the effect of assignment to intervention or the effect of starting and adhering to intervention). | Low Risk. The MIS-TLIF was applied properly and with consistency. |
| **Bias due to missing data:**  Bias that arises when later follow-up is missing for individuals initially included and followed (such as differential loss to follow-up that is affected by prognostic factors); bias due to exclusion of individuals with missing information about intervention status or other variables such as confounders. | Low Risk. The data reported for postoperative primary, and second outcome were comprehensive. |
| **Bias in measurement of outcomes:**  Bias introduced by either differential or non-differential errors in measurement of outcome data. Such bias can arise when outcome assessors are aware of intervention status, if different methods are used to assess outcomes in different intervention groups, or if measurement errors are related to intervention status or effects. | Low Risk. The data reported for postoperative primary, and second outcome were comprehensive. |
| **Bias in selection of the reported result:**  Selective reporting of results in a way that depends on the findings and prevents the estimate from being included in a meta-analysis (or other synthesis). | Low Risk. Data were reported for all patients. |
| **Overall risk of bias judgement** | Low Risk. This study included domains of the same risk for bias. |

| **Wang Z et al.** | |
| --- | --- |
| **Domain** | **Consensus** |
| **Bias Due to Confounding:**  Baseline confounding occurs when one or more prognostic variables (factors that predict the outcome of interest) also predicts the intervention received at baseline. | Moderate Risk. Analysis did not adjust for important confounder of baseline operation level. |
| **Bias in selection of participants into the study:**  When exclusion of some eligible participants, or the initial follow-up time of some participants, or some outcome events is related to both intervention and outcome, there will be an association between interventions and outcome even if the effects of the interventions are identical. | Low Risk. A broad variety of patients were included, with no potentially biasing screening steps. |
| **Bias in classification of interventions:**  Bias introduced by either differential or non-differential misclassification of intervention status. Non-differential misclassification is unrelated to the outcome and will usually bias the estimated effect of intervention towards the null. Differential misclassification occurs when misclassification of intervention status is related to the outcome or the risk of the outcome and is likely to lead to bias. | Low Risk. The surgical procedure of RA-TLIF is similar to the FG-TLIF. |
| **Bias due to deviations from intended interventions:**  Bias that arises when there are systematic differences between experimental intervention and comparator groups in the care provided, which represent a deviation from the intended intervention(s). Assessment of bias in this domain will depend on the type of effect of interest (either the effect of assignment to intervention or the effect of starting and adhering to intervention). | Low Risk. The TLIF was applied properly and with consistency. |
| **Bias due to missing data:**  Bias that arises when later follow-up is missing for individuals initially included and followed (such as differential loss to follow-up that is affected by prognostic factors); bias due to exclusion of individuals with missing information about intervention status or other variables such as confounders. | Low Risk. The data reported for postoperative primary, and second outcome were comprehensive. |
| **Bias in measurement of outcomes:**  Bias introduced by either differential or non-differential errors in measurement of outcome data. Such bias can arise when outcome assessors are aware of intervention status, if different methods are used to assess outcomes in different intervention groups, or if measurement errors are related to intervention status or effects. | Low Risk. The data reported for postoperative primary, and second outcome were comprehensive. |
| **Bias in selection of the reported result:**  Selective reporting of results in a way that depends on the findings and prevents the estimate from being included in a meta-analysis (or other synthesis). | Low Risk. Data were reported for all patients. |
| **Overall risk of bias judgement** | Moderate Risk. No single domain was rated as having a risk of bias greater than Moderate. |

| **Zhao XF et al.** | |
| --- | --- |
| **Domain** | **Consensus** |
| **Bias Due to Confounding:**  Baseline confounding occurs when one or more prognostic variables (factors that predict the outcome of interest) also predicts the intervention received at baseline. | Low Risk. No confounders were reported for each group. |
| **Bias in selection of participants into the study:**  When exclusion of some eligible participants, or the initial follow-up time of some participants, or some outcome events is related to both intervention and outcome, there will be an association between interventions and outcome even if the effects of the interventions are identical. | Moderate Risk. Unclear how many, if any, patients were eliminated during the pre-screening process due to comorbidities. The patients who died/withdrew consent were not included in analysis. |
| **Bias in classification of interventions:**  Bias introduced by either differential or non-differential misclassification of intervention status. Non-differential misclassification is unrelated to the outcome and will usually bias the estimated effect of intervention towards the null. Differential misclassification occurs when misclassification of intervention status is related to the outcome or the risk of the outcome and is likely to lead to bias. | Low Risk. The surgical procedure of RA-TLIF is similar to the FG-TLIF. |
| **Bias due to deviations from intended interventions:**  Bias that arises when there are systematic differences between experimental intervention and comparator groups in the care provided, which represent a deviation from the intended intervention(s). Assessment of bias in this domain will depend on the type of effect of interest (either the effect of assignment to intervention or the effect of starting and adhering to intervention). | Low Risk. The TLIF was applied properly and with consistency. |
| **Bias due to missing data:**  Bias that arises when later follow-up is missing for individuals initially included and followed (such as differential loss to follow-up that is affected by prognostic factors); bias due to exclusion of individuals with missing information about intervention status or other variables such as confounders. | Moderate Risk. Revision case was assessed out to post-op year 5 and assessed annually. However, patients who died were not factored into the analysis. |
| **Bias in measurement of outcomes:**  Bias introduced by either differential or non-differential errors in measurement of outcome data. Such bias can arise when outcome assessors are aware of intervention status, if different methods are used to assess outcomes in different intervention groups, or if measurement errors are related to intervention status or effects. | Low Risk. The data reported for postoperative primary, and second outcome were comprehensive. |
| **Bias in selection of the reported result:**  Selective reporting of results in a way that depends on the findings and prevents the estimate from being included in a meta-analysis (or other synthesis). | Low Risk. Data were reported for all patients. |
| **Overall risk of bias judgement** | Moderate Risk. No single domain was rated as having a risk of bias greater than Moderate. |

| **Mao JP et al.** | |
| --- | --- |
| **Domain** | **Consensus** |
| **Bias Due to Confounding:**  Baseline confounding occurs when one or more prognostic variables (factors that predict the outcome of interest) also predicts the intervention received at baseline. | Low Risk. No confounders were reported for each group. |
| **Bias in selection of participants into the study:**  When exclusion of some eligible participants, or the initial follow-up time of some participants, or some outcome events is related to both intervention and outcome, there will be an association between interventions and outcome even if the effects of the interventions are identical. | Low Risk. The patients who died/withdrew consent were not included in analysis. |
| **Bias in classification of interventions:**  Bias introduced by either differential or non-differential misclassification of intervention status. Non-differential misclassification is unrelated to the outcome and will usually bias the estimated effect of intervention towards the null. Differential misclassification occurs when misclassification of intervention status is related to the outcome or the risk of the outcome and is likely to lead to bias. | Low Risk. The surgical procedure of RA-TLIF is similar to the FG-TLIF. |
| **Bias due to deviations from intended interventions:**  Bias that arises when there are systematic differences between experimental intervention and comparator groups in the care provided, which represent a deviation from the intended intervention(s). Assessment of bias in this domain will depend on the type of effect of interest (either the effect of assignment to intervention or the effect of starting and adhering to intervention). | Low Risk. The TLIF was applied properly and with consistency. |
| **Bias due to missing data:**  Bias that arises when later follow-up is missing for individuals initially included and followed (such as differential loss to follow-up that is affected by prognostic factors); bias due to exclusion of individuals with missing information about intervention status or other variables such as confounders. | Low Risk. The data reported for postoperative primary, and second outcome were comprehensive. |
| **Bias in measurement of outcomes:**  Bias introduced by either differential or non-differential errors in measurement of outcome data. Such bias can arise when outcome assessors are aware of intervention status, if different methods are used to assess outcomes in different intervention groups, or if measurement errors are related to intervention status or effects. | Low Risk. The data reported for postoperative primary, and second outcome were comprehensive. |
| **Bias in selection of the reported result:**  Selective reporting of results in a way that depends on the findings and prevents the estimate from being included in a meta-analysis (or other synthesis). | Low Risk. Data were reported for all patients. |
| **Overall risk of bias judgement** | Low Risk. This study included domains of the same risk for bias. |

| **Jiang SD et al.** | |
| --- | --- |
| **Domain** | **Consensus** |
| **Bias Due to Confounding:**  Baseline confounding occurs when one or more prognostic variables (factors that predict the outcome of interest) also predicts the intervention received at baseline. | Low Risk. No confounders were reported for each group. |
| **Bias in selection of participants into the study:**  When exclusion of some eligible participants, or the initial follow-up time of some participants, or some outcome events is related to both intervention and outcome, there will be an association between interventions and outcome even if the effects of the interventions are identical. | Moderate Risk. Unclear how many, if any, patients were eliminated during the pre-screening process due to comorbidities. |
| **Bias in classification of interventions:**  Bias introduced by either differential or non-differential misclassification of intervention status. Non-differential misclassification is unrelated to the outcome and will usually bias the estimated effect of intervention towards the null. Differential misclassification occurs when misclassification of intervention status is related to the outcome or the risk of the outcome and is likely to lead to bias. | Low Risk. The surgical procedure of RA-MISTLIF is similar to the FG-MISTLIF. |
| **Bias due to deviations from intended interventions:**  Bias that arises when there are systematic differences between experimental intervention and comparator groups in the care provided, which represent a deviation from the intended intervention(s). Assessment of bias in this domain will depend on the type of effect of interest (either the effect of assignment to intervention or the effect of starting and adhering to intervention). | Low Risk. The MIS-TLIF was applied properly and with consistency. |
| **Bias due to missing data:**  Bias that arises when later follow-up is missing for individuals initially included and followed (such as differential loss to follow-up that is affected by prognostic factors); bias due to exclusion of individuals with missing information about intervention status or other variables such as confounders. | Low Risk. The data reported for postoperative primary, and second outcome were comprehensive. |
| **Bias in measurement of outcomes:**  Bias introduced by either differential or non-differential errors in measurement of outcome data. Such bias can arise when outcome assessors are aware of intervention status, if different methods are used to assess outcomes in different intervention groups, or if measurement errors are related to intervention status or effects. | Low Risk. The data reported for postoperative primary, and second outcome were comprehensive. |
| **Bias in selection of the reported result:**  Selective reporting of results in a way that depends on the findings and prevents the estimate from being included in a meta-analysis (or other synthesis). | Low Risk. Data were reported for all patients. |
| **Overall risk of bias judgement** | Moderate Risk. No single domain was rated as having a risk of bias greater than Moderate. |

| **Cui GY et al.** | |
| --- | --- |
| **Domain** | **Consensus** |
| **Bias Due to Confounding:**  Baseline confounding occurs when one or more prognostic variables (factors that predict the outcome of interest) also predicts the intervention received at baseline. | Low Risk. No confounders were reported for each group.. |
| **Bias in selection of participants into the study:**  When exclusion of some eligible participants, or the initial follow-up time of some participants, or some outcome events is related to both intervention and outcome, there will be an association between interventions and outcome even if the effects of the interventions are identical. | Moderate Risk. 11 of patients refused consent, and 7 patients dropped out after enrollment. |
| **Bias in classification of interventions:**  Bias introduced by either differential or non-differential misclassification of intervention status. Non-differential misclassification is unrelated to the outcome and will usually bias the estimated effect of intervention towards the null. Differential misclassification occurs when misclassification of intervention status is related to the outcome or the risk of the outcome and is likely to lead to bias. | Low Risk. The surgical procedure of RA-MISTLIF is similar to the FG-MISTLIF. |
| **Bias due to deviations from intended interventions:**  Bias that arises when there are systematic differences between experimental intervention and comparator groups in the care provided, which represent a deviation from the intended intervention(s). Assessment of bias in this domain will depend on the type of effect of interest (either the effect of assignment to intervention or the effect of starting and adhering to intervention). | Low Risk. The MIS-TLIF was applied properly and with consistency. |
| **Bias due to missing data:**  Bias that arises when later follow-up is missing for individuals initially included and followed (such as differential loss to follow-up that is affected by prognostic factors); bias due to exclusion of individuals with missing information about intervention status or other variables such as confounders. | Low Risk. The data reported for postoperative primary, and second outcome were comprehensive. |
| **Bias in measurement of outcomes:**  Bias introduced by either differential or non-differential errors in measurement of outcome data. Such bias can arise when outcome assessors are aware of intervention status, if different methods are used to assess outcomes in different intervention groups, or if measurement errors are related to intervention status or effects. | Low Risk. The data reported for postoperative primary, and second outcome were comprehensive. |
| **Bias in selection of the reported result:**  Selective reporting of results in a way that depends on the findings and prevents the estimate from being included in a meta-analysis (or other synthesis). | Low Risk. Data were reported for all patients. |
| **Overall risk of bias judgement** | Moderate Risk. No single domain was rated as having a risk of bias greater than Moderate. |
