# Supplementary material for: Comparison of robot-assisted versus fluoroscopy-guided transforaminal lumbar interbody fusion (TLIF) for lumbar degenerative diseases: a systematic review and meta-analysis of randomized controlled trails and cohort studies
Source: Syst Rev. 2024 Jul 5;13:170. doi: 10.1186/s13643-024-02600-6 (PMC11227242; doi:10.1186/s13643-024-02600-6)
Supplement: Supplementary file 2 — Supplementary Material 2. [file 13643_2024_2600_MOESM2_ESM.docx]

**Comparison of Robot-Assisted Versus Fluoroscopy-Guided Transforaminal Lumbar Interbody Fusion (TLIF) for Lumbar Degenerative Diseases: A Systematic Review and Meta-Analysis of Randomized Controlled Trails and Cohort Studies Protocol**

**OBJECTIVES**

The purpose of this study is to investigate whether the RA TLIF is superior to FG TLIF in treating lumbar degenerative disease.

**Primary and Secondary objective**

To determine whether in patients with lumbar degenerative disease, such as spondylolisthesis, lumbar spinal stenosis, RA TLIF is superior to FG TLIF in terms of the outcomes listed below:

- - Accuracy of percutaneous pedicle screw placement and proximal facet joint violation
  - Perioperative parameter such as radiation exposure, duration of surgery and EBL
  - Other outcome such as revision case

**METHODS**

**Study selection**

Inclusion criteria:

- Study design: All randomized controlled trials, prospective and retrospective cohort studies
- Population of interest: patients with lumbar degenerative disease, such as spondylolisthesis, lumbar spinal stenosis
- Interventions: Robot-assisted TLIF
- Comparator: Fluoroscopy-guided TLIF
- Outcomes:
  - Accuracy of percutaneous pedicle screw placement and proximal facet joint violation.
  - Perioperative parameter such as radiation exposure, duration of surgery and EBL.
  - Other outcome such as revision case.

Exclusion criteria:

- Studies lacking components mentioned above
- studies with insufficient data
- cadaveric and animal studies
- sample size per arm <10 participants.

**Data abstraction**

The following information will be collected where available:

- Name of trial/author/journal
- Year of publication
- Participants and Surgery
- The type of Robot System
- Sample size
- Characteristics of study participants (Age, Sex)
- Type of adherence intervention
- Control group mode of treatment
- Study results based on the outcomes of interest.
- Study design

**Search strategy**

Literature search will involve PubMed, EMbase, Web of science, CNKI, WanFang, VIP and the Cochrane library and grey literature via searching references of published systematic reviews. No language restriction was applied.

**Analysis plan**

- When two or more studies of an intervention are available that report on the same outcome of interest, the results will be pooled.
- Random- or common-effect model (also referred to as the fixed-effect model) were used, depending on studies heterogeneity.
- All analyses will be conducted in R.
- Statistical heterogeneity was assessed with the Q-test and the I2 statistic. I2 values of 25%, 50%, and 75% were considered to indicate low, moderate, and high heterogeneity, respectively.
- If >10 studies are available for an intervention, funnel plots will be used to assess publication bias.
- Quality assessment
  - The Cochrane Risk of Bias tool will be used to assess the quality of all randomized controlled trials.
  - The Newcastle Ottawa Scale will be used to assess the quality of cohort studies.

**Data management**

The initial data analysis will address only the objectives specified above. Preliminary findings from these analyses will be circulated among contributing investigators for their comments and suggestions about further analysis. Any additional analysis will be proposed to all investigators, although as long as related to the objectives above we will not seek unanimous approval of any such additional analyses. However, objections to the new analysis will be addressed and resolved before proceeding. (In other words, if a collaborator does not respond, that is taken as implicit approval). If we think of analyses to address entirely different and novel objectives that are not considered or foreseen here, we will seek approval from ALL investigators before embarking on any such analyses.

All proposed publications will be reviewed and approved by all investigators before public presentation or submission for publication. The authorship will include all responsible investigators contributing data.

Two investigators independently selected articles according to the criteria described above. The full text was scanned to determine whether articles fit the inclusion criteria. We resolved disagreements by discussion until a consensus was search. If no consensus was reached, a third investigator was consulted.
